# Immunological Strategies in Gastric Cancer: How Toll-like Receptors 2, -3, -4, and -9 on Monocytes and Dendritic Cells Depend on Patient Factors?

**DOI:** 10.3390/cells13201708

**Published:** 2024-10-16

**Authors:** Marek Kos, Krzysztof Bojarski, Paulina Mertowska, Sebastian Mertowski, Piotr Tomaka, Łukasz Dziki, Ewelina Grywalska

**Affiliations:** 1 Department of Public Health, Medical University of Lublin, 1 Chodźki Street, 20-093 Lublin, Poland; marek.kos@umlub.pl; 2 General Surgery Department, SP ZOZ in Leczna, 52 Krasnystawska Street, 21-010 Leczna, Poland; k.bojarski@szpital.leczna.pl; 3 Department of Experimental Immunology, Medical University of Lublin, 4a Chodźki Street, 20-093 Lublin, Poland; paulina.mertowska@umlub.pl (P.M.); ewelina.grywalska@umlub.pl (E.G.); 4 Department of Anesthesiology and Intensive Care, SP ZOZ in Leczna, 52 Krasnystawska Street, 21-010 Leczna, Poland; p.tomaka@szpital.leczna.pl; 5 Department of General and Oncological Surgery, Faculty of Medicine, Medical University of Lodz, 251 Street, 92-213 Lodz, Poland; lukasz.dziki@umed.lodz.pl

**Keywords:** TLR, stage, gender, age, gastric cancer, immune system

## Abstract

(1) Introduction: Toll-like receptors (TLRs) are key in immune response by recognizing pathogen-associated molecular patterns (PAMPs) and damage-associated molecular patterns (DAMPs). In gastric cancer (GC), TLR2, TLR3, TLR4, and TLR9 are crucial for modulating immune response and tumor progression. (2) Objective: This study aimed to assess the percentage of dendritic cells and monocytes expressing TLR2, TLR3, TLR4, and TLR9, along with the concentration of their soluble forms in the serum of GC patients compared to healthy volunteers. Factors such as disease stage, tumor type, age, and gender were also analyzed. (3) Materials and Methods: Blood samples from newly diagnosed GC patients and healthy controls were immunophenotyped using flow cytometry to assess TLR expression on dendritic cell subpopulations and monocytes. Serum-soluble TLRs were measured by ELISA. Statistical analysis considered clinical variables such as tumor type, stage, age, and gender. (4) Results: TLR expression was significantly higher in GC patients, except for TLR3 on classical monocytes. Soluble forms of all TLRs were elevated in GC patients, with significant differences based on disease stage but not tumor type, except for serum TLR2, TLR4, and TLR9. (5) Conclusions: Elevated TLR expression and soluble TLR levels in GC patients suggest a role in tumor pathogenesis and progression, offering potential biomarkers and therapeutic targets.

## 1. Introduction

Gastric cancer (GC) remains one of the most common cancers globally, being the fourth most common in men and seventh in women [1]. The number of new cases is expected to rise by 63%, reaching 1.8 million by 2040, with deaths possibly increasing to 1.3 million [1,2]. Key risk factors include infection with *Helicobacter pylori*, smoking, and a diet high in salt and processed foods [2,3]. Symptoms, when present, may include abdominal pain, nausea, difficulty swallowing, and unexplained weight loss. Treatment options depend on the stage and location of the cancer and may involve surgery, chemotherapy, and other targeted therapies. In the face of the increasing number of cases and difficulties in early diagnosis of GC, it is necessary to intensify research on new etiological and diagnostic factors. Understanding these processes may contribute to the development of more effective methods of detecting and treating gastric cancer, which is crucial in reducing the morbidity and mortality associated with this tumor.

In recent years, there has been growing interest in studying the role of different subpopulations of immune cells in the tumor microenvironment. Understanding the mechanisms by which these cells influence the development and progression of GC has become particularly important. Among these cells, monocytes and dendritic cells (DCs) are important. Monocytes, precursors of macrophages and DCs, play an important role in initiating the immune response. In GC, they can differentiate into tumor-associated macrophages (TAMs), which promote tumor development by stimulating angiogenesis, immunosuppression, and tumor cell invasion [4,5,6,7]. In the context of GC, three main monocyte subpopulations can be distinguished: classical, nonclassical, and intermediate. Classical monocytes (CD14++CD16−) play a fundamental role in the inflammatory response and are recruited to sites of tissue damage [8,9,10]. Non-classical monocytes (CD14+CD16++) act as “guardians” of blood vessels, maintaining their integrity and participating in regenerative processes, and also play an important role in the antiviral response [8,11]. Intermediate monocytes (CD14+CD16+) combine features of both previous subpopulations, acting as a bridge between the inflammatory response and repair processes [9,12]. In cancers, including GC, changes in the number and function of these subpopulations are observed. Studies have shown that patients with advanced gastric cancer have an increased number of classical monocytes in the circulation, which correlates with a poorer prognosis [13,14]. In addition, these monocytes can undergo phenotypic changes, promoting the creation of an immunosuppressive microenvironment that supports tumor development through the secretion of angiogenic factors such as VEGF [13,15,16]. Suppression of T cell responses enables cancer cells to evade immune surveillance, which further accelerates disease progression [13,17]. Dendritic cells also play an important role, as they are key players in antigen presentation and initiation of T cell responses. In the context of GC, two subpopulations of DC are of particular importance: BDCA-1+ (CD1c+) and BDCA-2+ (CD303+) [18,19,20,21]. BDCA-2+ cells are plasmacytoid dendritic cells (pDC), which are normally responsible for the production of type I interferon in response to viral infections [18,20,21].

In GC, BDCA-1+ and BDCA-2+ cells may play a dual role. On the one hand, they support the antitumor response by presenting tumor antigens to T lymphocytes, which promotes immune activation [22,23,24]. On the other hand, in certain situations, they may promote immunosuppression, especially when regulatory T lymphocytes (Tregs) are recruited in the tumor microenvironment, which limits the effectiveness of the immune response against the tumor [22,25,26]. Toll-like receptors (TLRs), expressed on both immune and tumor cells, play a key role in GC. TLR-2, TLR-3, TLR-4, and TLR-9 are important in recognizing pathogen-associated molecular patterns (PAMPs) and damage-related signals (DAMPs) [27,28,29]. TLR-2 recognizes bacterial lipoproteins, activating immune cells via the MyD88 pathway, leading to the production of proinflammatory cytokines and the recruitment of effector cells. In GC, TLR-2 can enhance antitumor responses by increasing CD8+ T cell cytotoxicity but can also promote immunosuppression [28,30,31]. TLR-4 detects lipopolysaccharide (LPS) from *Helicobacter pylori*, initiating the MyD88 and TRIF pathways and promoting chronic inflammation, potentially leading to carcinogenesis [32,33,34]. TLR-3 recognizes double-stranded RNA and induces antiviral responses, potentially promoting tumor cell apoptosis, although its role in GC requires further investigation [35,36,37]. TLR-9, which detects unmethylated CpG DNA sequences, stimulates the production of type I interferon and proinflammatory cytokines, enhancing antitumor responses. However, in advanced GC, TLR-9 activation may promote immunosuppression [38,39,40]. It is worth emphasizing that previous studies show that TLR-related pathways can play both anti-tumor and pro-tumor effects, and in some cases even a dual role in cancer cells, making them molecules with potential as biomarkers or prognostic factors [41,42]. At the same time, TLR agonists may play an essential role in cancer immunotherapy by activating the innate immune response and supporting adaptive immunity, particularly in gastric cancer. For example, TLR9 agonists, such as CpG-ODN, activate dendritic cells and macrophages, stimulating T lymphocytes for anti-cancer activity. TLR7/8 agonists, such as R848, are effective in combination with checkpoint inhibitors. Additionally, TLR2/TLR4 agonists are used to treat bladder cancer [43,44].

In light of the above reports, continuing research on the role of monocytes and DCs in the pathogenesis of GC is crucial for developing new therapeutic strategies. Our study aimed to assess the percentage of monocytes and DCs expressing TLR-2, TLR-3, TLR-4, and TLR-9 receptors and their soluble forms in serum in the context of clinical parameters of GC patients.

## 2. Materials and Methods

### 2.1. Characteristics of Patients Included in the Study

A total of 69 patients with newly diagnosed, untreated gastric cancer and 30 healthy volunteers were included in this study. Detailed inclusion and exclusion criteria for patients in the individual study groups are presented below. The inclusion criteria for patients included in the study include a new diagnosis of gastric cancer, where patients must be newly diagnosed and must not have previously received any anticancer treatment, such as chemotherapy, radiotherapy, or surgery. Patients with any stage of disease (from stage I to IV) confirmed by histopathology can be included in the study. Patients must be over 18 years of age, and all participants must provide written, informed consent to participate in the study. Additionally, patients must have a sufficient white blood cell count and other hematological parameters within the normal range to allow for blood sampling for analysis. Exclusion criteria include previous gastric cancer treatment, the presence of another active malignancy, and participation in clinical trials. Patients with confirmed immunological disorders, such as autoimmune diseases and those taking immunosuppressive medications are also excluded. Patients with active viral, bacterial, or fungal infections are also ineligible for the study, as are pregnant or breastfeeding women due to the potential risk to the mother and child and changes in the immune system associated with pregnancy. Additionally, patients in poor general condition, such as an ECOG score of 2 or higher, are excluded from the study because they may not be able to meet the study requirements. Lack of informed consent also excludes the patient from participation, as does the presence of severe, decompensated diseases, such as heart failure, renal failure, or liver failure, which may affect the results of the study or the patient’s safety.

The criteria for including healthy volunteers in the study include no history of cancer, good general health, and age over 18 years. Volunteers must be free from chronic diseases such as cardiovascular disease, diabetes, or chronic respiratory diseases that could affect the study results. Additionally, volunteers must not be taking immunosuppressive drugs or other drugs that affect the immune system, and they must not be carriers of chronic infections such as viral hepatitis or HIV. It is also necessary for volunteers to give written, informed consent to participate in the study, including blood sampling. Exclusion criteria include the presence of autoimmune diseases such as rheumatoid arthritis or systemic lupus erythematosus, taking immunosuppressive drugs, recent vaccinations (in the last 4 weeks), and the presence of an acute infection at the time of recruitment. Pregnant or breastfeeding women are also excluded due to changes in the immune system associated with pregnancy and lactation. In addition, volunteers with chronic medical conditions requiring ongoing treatment that may affect the immune system are not eligible for the study. Finally, individuals who have participated in other clinical trials in the past 3 months are excluded to avoid interference with the results.

Blood samples (from the basilic vein) were collected from each participant for immunological analyses as part of the study procedure. Blood collection was performed using standard techniques with an aseptic technique. First, 10 mL of blood was collected into tubes containing EDTA, which acts as an anticoagulant, preventing blood clotting and allowing the collection of plasma and mononuclear cells (such as monocytes and dendritic cells) for further analysis. Additionally, 5 mL of blood was collected into tubes intended for serum. These tubes did not contain anticoagulants, which allowed the blood to clot naturally. After collection, the blood was left to coagulate at room temperature for approximately 30 min, and then the tubes were centrifuged to separate the serum from the morphological elements of the blood. The obtained serum was then separated and stored under appropriate conditions until biochemical analyses were performed. In our study, a serological test was used to assess *H. pylori* infection in patients with GC. Three classes of antibodies were determined: IgG, IgA, and IgM. IgG antibodies are the most commonly used marker because they appear several weeks after infection and persist for a long time, which allows for the detection of both active and past infection. Increased IgG concentration may indicate chronic infection or cured infection. In turn, IgA antibodies are associated with a more acute phase of infection, and their presence may suggest active infection of the gastric mucosa. On the other hand, IgM antibodies, which appear at an early stage of infection, indicate a fresh infection, but in our study, their determination was not crucial because *H. pylori* infections in gastric cancer are usually chronic. Of the 69 patients recruited to the study, only 7 patients had a positive serological test result for IgG antibodies against *H. pylori*. Patients in the control group were negative for *H. pylori*. This means that most patients did not have an active infection with the bacteria, which minimizes the influence of *H. pylori* on the results regarding TLR expression and immune response in this group.

### 2.2. Immunophenotyping Procedure and Instrumentation for Peripheral Blood Analysis

The material used for immunophenotyping was whole peripheral blood. The sample was incubated with monoclonal antibodies: CD14 FITC; CD16 PerCP 5.5; HLA-DR APC-CY7; CD11c BUV395; CD123 BV510; CD1c BV650; CD303 BV605; CD141 BB700; HLA-DR APC-CY7; anti-TLR2 APC; anti-TLR4 PE; anti-TLR3; anti-TLR9 APC (BD, Franklin Lakes, NJ, USA, and Biolegend, San Diego, CA, USA). To obtain better signal quality, BD Horizon™ Brilliant Stain Buffer (BD, Franklin Lakes, NJ, USA) was also used. Samples were then lysed to remove red blood cells and then washed with BD Pharmingen™ Stain Buffer (BSA). The BD Cytofix/Cytoperm™ Fixation/Permeabilization Kit was used to assess intracellular molecules. Samples were analyzed using CytoFLEX LX (Beckman Coulter, Indianapolis, IN, USA), and data were analyzed using Kaluza Analysis v2.1 software (sample analysis in Supplementary Materials Figure S1). To ensure instrument performance, the instrument was evaluated using CytoFLEX Ready to Use Daily QC Fluorosphere reagents (Beckman Coulter, Indianapolis, IN, USA).

### 2.3. Assessment of Soluble TLR-2, TLR-3, TLR-4, and TLR-9 Levels in Serum by ELISA

Soluble forms of TLR-2, TLR-3, TLR-4, and TLR-9 receptors in the patients’ serum were assessed by enzyme-linked immunosorbent assay using commercial kits. The kits used had the following sensitivities: TLR-2 (15.6–1000 pg/mL), TLR-3 (31.2–2000 pg/mL), TLR-4 (15.6–1000 pg/mL), and TLR-9 (31.2–2000 pg/mL) (R&D Systems, Minneapolis, MN, USA). Measurement was performed on a Victor 4.0 microplate reader (PerkinElmer, Hong Kong, China). The results were analyzed using a calibration curve obtained from standards, which allowed the determination of concentrations of individual TLR-2, TLR-3, TLR-4, and TLR-9 in serum samples.

### 2.4. Statistical Analysis and Data Visualization Methods

Statistical evaluation was performed using Tibco Statistica 13.3 software in Palo Alto, CA, USA, and graph visualization was performed using GraphPad Prism Software v. 9.4.1. San Diego, CA, USA. The Shapiro-Wilk test was used to analyze the data and check for normal distribution. Although this test is parametric, it was used to verify that the data met the assumptions necessary for the use of parametric methods. Nonparametric methods were used where the data did not meet these assumptions. The Mann-Whitney U test was used to compare two independent groups, which tests whether there are statistically significant differences between the medians of these groups, which is especially useful for small samples and data that are not normally distributed. When more than two independent groups were compared, the Kruskal-Wallis test, an extension of the Mann-Whitney U test, was used to assess whether at least one group differed significantly in terms of the median from the others. The Kruskal-Wallis test is a nonparametric equivalent of the one-way analysis of variance (ANOVA). Additionally, a version of the Kruskal-Wallis test with a Bonferroni correction was used to adjust the level of significance for multiple comparisons, which improved the accuracy of the results. Spearman’s rank correlation was used to assess the relationship between variables. This nonparametric statistical method measures the strength and direction of a monotonic relationship between two variables. The Spearman test is particularly useful for data that are not normally distributed or are on an ordinal scale. The Spearman rank correlation result allows you to assess whether there is a statistically significant correlation between the analyzed parameters and determine whether this relationship is positive or negative.

## 3. Results

### 3.1. Demographic and Clinical Analysis of the Study Patients

**Figure 1:**
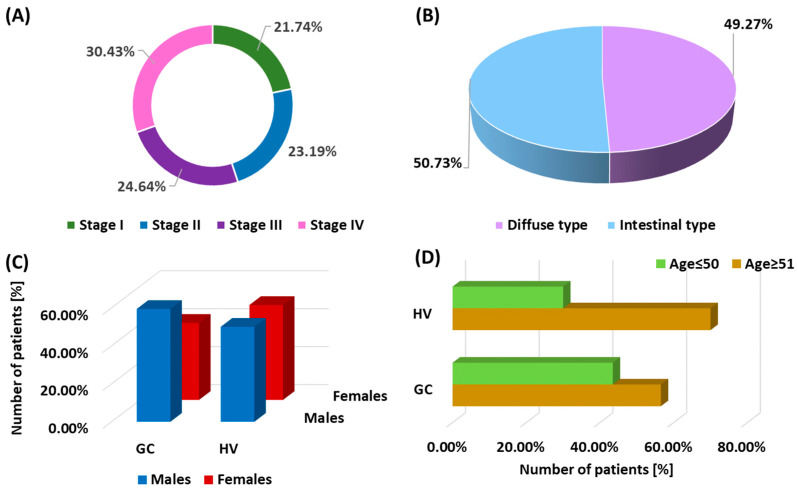
Graphical representation of the characteristics of GC patients and healthy volunteers included in the study. (**A**) illustrates the distribution of GC patients by disease stage at diagnosis; (**B**) shows the division of patients by histopathological type of GC: intestinal type and diffuse type; (**C**) presents the gender distribution in both the GC patient group and the healthy control group; and (**D**) shows the age diversity of the recruited GC patients and healthy volunteers, showing the full age range of the study subjects. Abbreviations: GC, gastric cancer; HV, healthy volunteers.

Based on detailed inclusion and exclusion criteria (described in the Section 2), 69 patients with histopathologically confirmed GC with a mean age of 60.28 ± 14.04 years (median: 58; range: 39–86) and 30 healthy volunteers with an age of 58.97 ± 12.65 years (median: 58.50; range: 37–82) were included in the study. Statistical analysis did not reveal any statistically significant differences in the age of the compared patient groups. Among the patients with GC, 21.74% were patients with stage I; 23.19% were patients with stage II; 24.64% were patients with stage III; and 30.43% were patients with stage IV at diagnosis (Figure 1A). In addition, 50.73% of patients were diagnosed with intestinal type and 49.27% with diffuse type (Figure 1B). The gender distribution in both the study group and healthy volunteers constituting the control group was 59.42% male and 40.58% female in patients with GC and 50% female and male in healthy volunteers (Figure 1C). Additionally, the age diversity of the recruited patients is presented in Figure 1D. All patients underwent basic analysis of peripheral blood morphology and assessment of the percentage of the examined immune cells, i.e., dendritic cells (BDCA-1 and BDCA-2), as well as monocytes (classical: CD14++CD16-, intermediate: CD14+CD16+, and non-classical: CD14+CD16++). Detailed data are presented in Table 1.

The comparisons conducted showed that statistically significant differences between the patients studied concerned an increase in the number of white blood cells and the percentage of intermediate and non-classical monocytes in peripheral blood in patients with GC compared to healthy volunteers. Moreover, the number of neutrophils and red blood cells, platelets, and hemoglobin, as well as the percentage of both studied dendritic cell populations in patients with GC compared to healthy volunteers, was significantly reduced.

### 3.2. Assessment of Differences in the Percentage of TLR-2, TLR-3, TLR-4, and TLR-9 on DCs and Monocytes and the Concentration of Soluble Forms in Serum between GC Patients and Healthy Volunteers

In the first stage of our study, we determined the percentage of selected TLRs on both DC and monocyte subpopulations, and the obtained data are presented in Table 2.

As a result of the presented data, the percentage of occurrence of almost all tested TLRs in individual immune cell subpopulations was significantly higher (except for TLR-3 in classical monocytes) in patients diagnosed with GC compared to healthy volunteers. These analyses were supplemented by the assessment of the concentration of soluble forms of the tested TLRs in the serum of all recruited patients. The obtained results showed an increase in the concentration of all soluble forms of the tested TLRs in patients with GC compared to healthy volunteers (Table 2). Due to the observed large ranges of the results of our studies in patients with GC, we decided to check in the further stages of the study whether these changes could be conditioned by individual characteristics of the patients, such as types of cancer, stage of the disease, gender, or age of the recruited patients.

### 3.3. Assessment of the Differences in the Percentage of TLR-2, TLR-3, TLR-4, and TLR-9 on DCs and Monocytes and the Concentration of Soluble Forms in Serum between Patients with GC Depending on the Type of Cancer and the Stage of the Disease

In the first stage, we compared the obtained results of the studies in terms of the assessment of changes in morphological and immunological parameters between patients with the diffuse and intestinal type and healthy volunteers (Supplementary Materials Table S1). We did not observe statistically significant changes between patients with the diffuse and intestinal type for all analyzed parameters, except for the concentration of soluble forms for TLR-2, TLR-4, and TLR-9 (Supplementary Materials Table S1). In the case of comparison of both diffuse and intestinal type with healthy individuals, almost all of the parameters studied were significantly higher in patients with GC (regardless of type) except for the percentage of classic monocytes and TLR-3+ on classic monocytes, as well as TLR-9+ on classic monocytes between intestinal type and healthy volunteers (Supplementary Materials Table S1).

We noted significantly more statistically significant differences when analyzing patients with GC with a detailed stage of their disease at diagnosis (Supplementary Materials Table S2).

**Figure 2:**
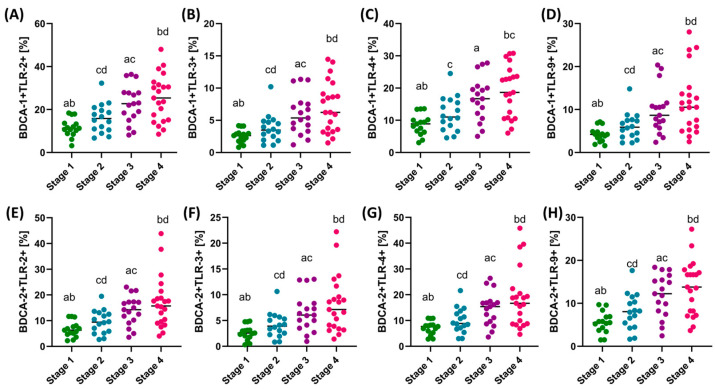
Comparison of the obtained results of the assessment of the percentage of the studied TLRs on dendritic cell subpopulations between patients in the individual stages of the disease. (**A**–**D**) Analysis of the percentage of TLR-2, TLR-3, TLR-4, and TLR-9 on BDCA-1 dendritic cells; (**E**–**H**) Analysis of the percentage of TLR-2, TLR-3, TLR-4, and TLR-9 on BDCA-2 dendritic cells. Statistically significant differences between the individual stages are marked with letters. To facilitate interpretation, the appropriate GC stages are marked with individual colors (green—stage I; blue—stage II; purple—stage III; pink—stage IV).

In the case of analysis of the percentage of the occurrence of the individual tested TLRs on the studied dendritic cell subpopulations, we can see that with the increasing stage of the disease, the percentage of cells showing positive expression of TLR-2, TLR-3, TLR-4, and TLR-9 increases. However, detailed analysis showed that statistically significant changes in the case of dendritic cells concern stages I and III, I and IV, II and III (except BDCA-1+ TLR4+), and II and IV (Figure 2). We did not observe statistically significant changes between patients in stages I and II and III and IV.

**Figure 3:**
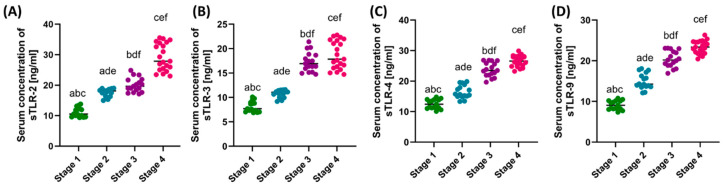
Comparison of the obtained results of the concentration of soluble forms of the studied TLRs in serum between patients in individual stages of the disease. (**A**–**D**) Analysis of serum concentrations of sTLR-2, sTLR-3, sTLR-4, and sTLR-9. Statistically significant differences between individual stages are marked with letters. To facilitate interpretation, the appropriate GC stages are marked with individual colors (green—stage I; blue—stage II; purple—stage III; pink—stage IV).

**Figure 4:**
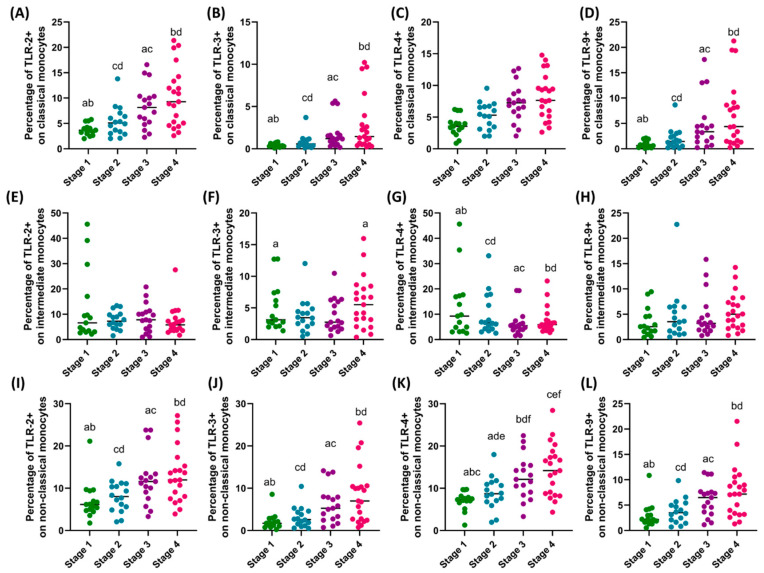
Comparison of the obtained results of the assessment of the percentage of the studied TLRs on monocyte subpopulations between patients in the individual stages of the disease. (**A**–**D**) Analysis of the percentage of TLR-2, TLR-3, TLR-4, and TLR-9 on classical monocytes; (**E**–**H**) Analysis of the percentage of TLR-2, TLR-3, TLR-4, and TLR-9 on intermediate monocytes; (**I**–**L**) Analysis of the percentage of TLR-2, TLR-3, TLR-4, and TLR-9 on non-classical monocytes. Statistically significant differences between individual stages are marked with letters. To facilitate interpretation, the appropriate GC stages are marked with individual colors (green—stage I; blue—stage II; purple—stage III; pink—stage IV).

In the case of the analyzed monocyte subpopulations, the same trends as in the case of dendritic cells can be noted for the percentage of TLRs examined on classical (except for TLR4+) and non-classical monocytes (except for TLR4+, which are also significant between I and II and III and IV) (Figure 3). In the case of the analysis of intermediate monocytes, a statistically significant relationship was only found in the differences between the percentage of TLR3+ in patients with stage I and IV (Figure 4).

Analysis of the concentration of soluble forms of the studied TLRs showed a similar trend as the immunophenotypic assessment. With the increasing disease advancement, the observed concentration of all analyzed TLRs increased. Moreover, statistical analysis confirmed that the obtained results are significantly different between all stages of GC (Figure 3).

### 3.4. Assessment of the Differences in the Percentage of TLR-2, TLR-3, TLR-4, and TLR-9 on DCs and Monocytes and the Concentration of Soluble Forms in Serum between Patients with GC Depending on Gender and Age

**Figure 5:**
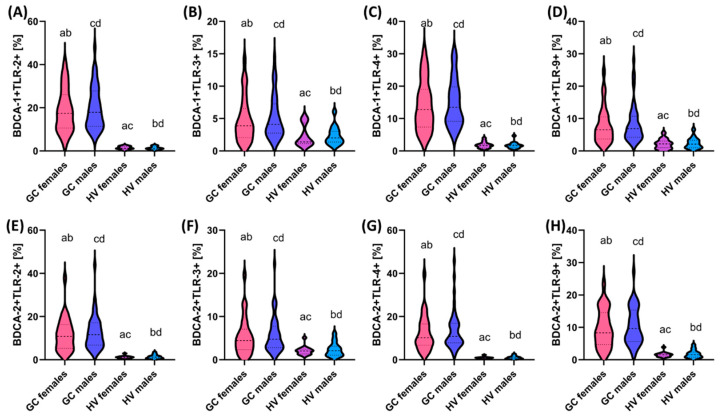
Comparison of the obtained results of the assessment of the percentage of the studied TLRs on dendritic cell subpopulations between patients depending on the patient’s gender. (**A**–**D**) Analysis of the percentage of TLR-2, TLR-3, TLR-4, and TLR-9 on BDCA-1 DCs; (**E**–H) Analysis of the percentage of TLR-2, TLR-3, TLR-4, and TLR-9 on BDCA-2 dendritic cells. Statistically significant differences between individual stages are marked with letters. To facilitate interpretation, the respective sexes are marked with individual colors (pink—women with GC; navy blue—men with GC; purple—women HV; light blue—men HV).

**Figure 6:**
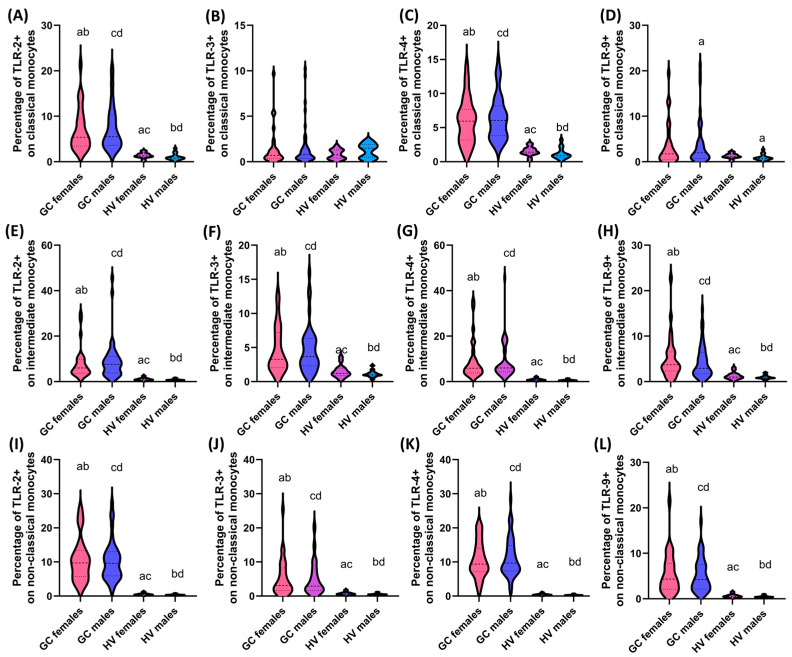
Comparison of the obtained results of the assessment of the percentage of the studied TLRs on monocyte subpopulations between patients depending on the patient’s gender. (**A**–**D**) Analysis of the percentage of TLR-2, TLR-3, TLR-4, and TLR-9 on classical monocytes; (**E**–H Analysis of the percentage of TLR-2, TLR-3, TLR-4, and TLR-9 on intermediate monocytes; (**I**–**L**) Analysis of the percentage of TLR-2, TLR-3, TLR-4, and TLR-9 on non-classical monocytes. Statistically significant differences between individual stages are marked with letters. To facilitate interpretation, the respective genders are marked with individual colors (pink—women with GC; navy blue—men with GC; purple—women HV; light blue—men HV).

**Figure 7:**
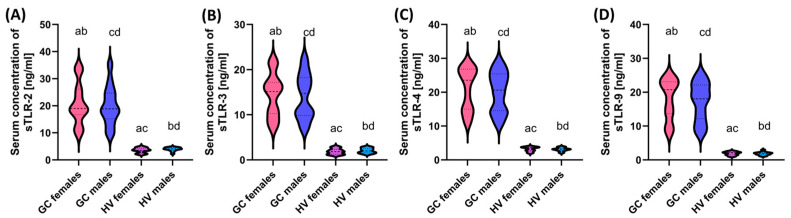
Comparison of the obtained results of the concentration of soluble forms of the studied TLRs in serum between patients, taking into account differences in gender. (**A**–**D**) Analysis of serum concentrations of sTLR-2, sTLR-3, sTLR-4, and sTLR-9. Statistically significant differences between individual stages are marked with letters. To facilitate interpretation, individual colors indicate the appropriate genders (pink—women with GC; navy blue—men with GC; purple—women HV; light blue—men HV).

In the next stage of our analyses, we decided to take a closer look at the variability of the obtained research results in terms of differences within the individual genders of the recruited patients and healthy volunteers. Detailed data are presented in Supplementary Materials Table S3. We did not observe statistically significant differences between women and men with GC, except for differences in the level of white blood cells (Supplementary Materials Table S3). In the case of the analyzed TLRs on individual cells of the immune system, the observed changes were not statistically significant (Figure 5, Figure 6 and Figure 7). Statistically significant changes were observed between women with GC and women from the group of healthy volunteers, especially in the percentage of DCs (BDCA-1 and BDCA-2) showing positive expression of all tested TLRs. The same changes were demonstrated in men with GC and men from the group of healthy volunteers, as well as between women with GC and healthy men and men with GC and healthy women (Figure 5).

In the case of analyses concerning the percentage of the studied TLRs on individual monocyte subpopulations, a similar trend as in the case of DCs was noted for the percentage of non-classical and intermediate monocytes showing positive expression of TLR-2, TLR-3, TLR-4, and TLR-9 (Figure 6). More diverse relationships were observed for the subpopulation of classical monocytes, where no statistically significant differences were noted for the expression of TLR-3 in all analyzed combinations of gender and TLR-9, except for men with GC and men from the group of healthy volunteers (Figure 6). Statistically significant relationships in the case of classical monocytes concerned the percentage of TLR-2 and TLR-4 between women with GC and healthy volunteers, women with GC and healthy men, and between men with GC and healthy men, and men with GC and healthy women (Figure 6).

Analyses comparing the levels of soluble TLRs between the sexes of recruited patients revealed statistically significant associations for TLR-2 and TLR-4, as well as TLR-3 and TLR-9, between women with GC and healthy volunteers; women with GC and healthy men; and between men with GC and healthy men and men with GC and healthy women (Figure 7).

The next step was to assess the variation of the obtained test results in terms of changes that occur with the age of the patients. For this purpose, we classified the recruited patients into people under 50 years of age (≤50) and people over 51 years of age (≥51). Literature data indicate that GC in people ≤50 years of age is much rarer than in older people, where the incidence increases with age. The etiology of GC in younger patients may be more related to genetic factors, while in older patients, environmental factors such as diet, smoking, *H. pylori* infection, and others play a greater role. Moreover, scientific studies indicate that in younger patients (≤50 years of age), GC often has different biological features than in older patients. This may include differences in histological subtypes, malignancy, or the frequency of genetic mutations. Younger people may be more likely to have cancer associated with genetic factors, such as hereditary cancer syndromes such as Lynch syndrome or *CDH1* mutations associated with hereditary diffuse gastric cancer.

In the group of patients ≤50 years, 30 patients with GC were classified (7 patients with stage I; 9 patients with stage II; 6 patients with stage III; and 8 patients with stage IV), of whom 56.67% had the diffuse type, while 43.33% had the intestinal type. In the group of patients with GC ≥51 years (8 patients with stage I; 7 patients with stage II; 11 patients with stage III; and 13 patients with stage IV), 46.15% had the diffuse type, while 53.84% had the intestinal type. In addition, the first group of patients included 13 women and 17 men, while the second group included 14 women and 25 men.

Detailed analysis of the obtained results of laboratory tests of morphology and immunophenotyping showed significant differences between GC patients ≤50 years and GC patients ≥51 years in the percentage of monocytes, and in particular the subpopulations: classical, intermediate, and non-classical, which were higher in older patients (Supplementary Materials Table S4). Statistically significant differences also concerned changes in the values of the tested TLR parameters between GC patients, which showed an increase in the percentage of the tested receptors on both tested subpopulations of DCs and classical and non-classical monocytes in older patients (Table 3). In the case of the assessment of the percentage of TLR-2, TLR-3, TLR-4, and TLR-9 on intermediate monocytes, no statistically significant differences were observed in their levels between both age groups (Table 3). Moreover, in almost all analyzed parameters we noted significant differences between GC patients and healthy volunteers ≥51 years (except for the percentage of TLR-3 on classical monocytes). In the case of GC patients and healthy volunteers ≤50 years, statistically significant changes did not concern the percentage of TLR-3 on BDCA-1 and BDCA-2; TLR-3 and TLR-9 on classical monocytes (Table 3). The statistical analysis of the results of the concentration of soluble forms of TLRs tested showed no significant differences between GC patients depending on the age group, but significant differences were already observed between GC patients and healthy volunteers ≥51 years and between GC patients and healthy volunteers ≤50 years.

### 3.5. Analysis of Correlations and ROC Curves of Obtained Test Results with Particular Consideration of Type, Stage, Age, and Gender of Recruited GC Patients

**Figure 8:**
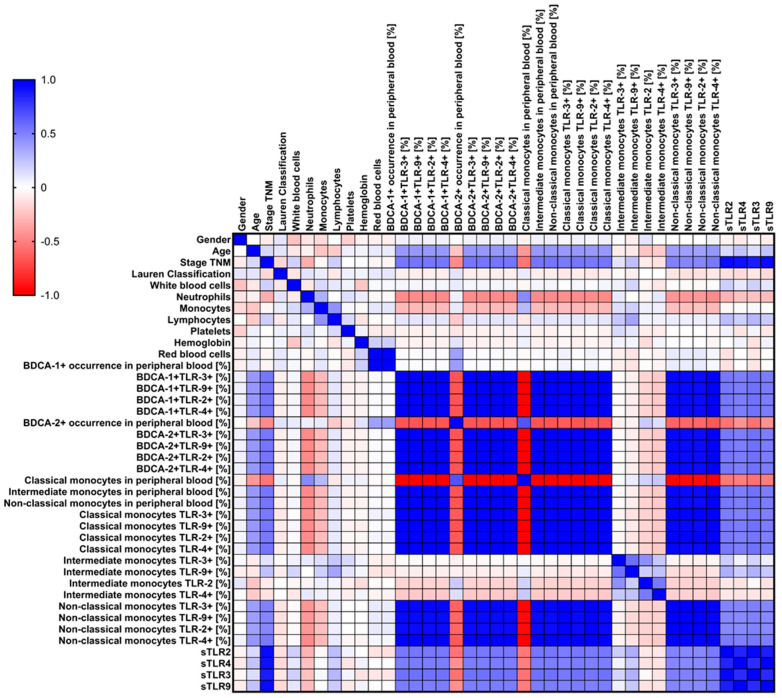
Schematic representation of the Spearman rank correlation of the studied parameters in patients with newly diagnosed GC. For ease of interpretation, negative correlations are marked in red, while positive correlations are marked in blue. The intensity of the coloring of individual pairs indicates the strength of correlations.

The analysis of correlations of the studied morphology and immunophenotype parameters conducted by our team, with particular consideration of the percentage of occurrence of the studied TLRs on individual subpopulations of dendritic cells and monocytes in the context of changes in individual characteristics (stage, type, gender, and age) of GC patients, showed several statistically significant relationships. We noted 91 negative correlations, of which 28.57% were low correlations; 34.07% were moderate correlations; 17.58% were high correlations; and 19.78% were full correlations (Supplementary Materials Table S5). We observed significantly more, as many as 283 positive correlations, of which 5.65% were low correlations; 38.52% moderate correlations; 0.35% high correlations; 1.77% very high correlations; and 53.71% full correlations (Supplementary Materials Table S5). Due to the amount of data obtained, detailed information is presented in Supplementary Materials Table S5 and graphically in Figure 8.

**Figure 9:**
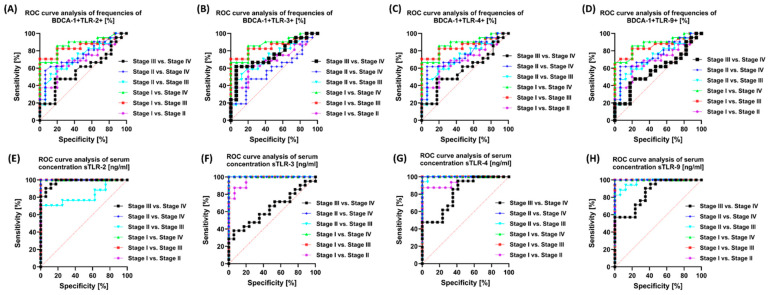
Graphical representation of selected ROC curves depending on the disease stage in GC patients. (**A**–**D**) ROC curves presenting a comparison of the sensitivity and specificity of the tested TLRs on BDCA-1; (**E**–**H**) ROC curves presenting a comparison of the sensitivity and specificity of the tested concentrations of soluble forms of TLRs in the serum of patients.

Due to the significance of the data obtained during our analyses, we decided to check in the further stages of our considerations whether the observed differences in the percentage of occurrence of individual TLRs on the examined immune cells and their serum concentrations could serve as potential molecules differentiating individual patients due to their disease stage, gender, and age. For this purpose, we performed a series of ROC curve analyses, paying attention to the parameters indicated above. First, we analyzed the sensitivity and specificity of the obtained test results in terms of their use to distinguish individual stages in patients with GC. Detailed data are presented in Supplementary Materials Table S6, while an example graphical representation is in Figure 9.

**Figure 10:**
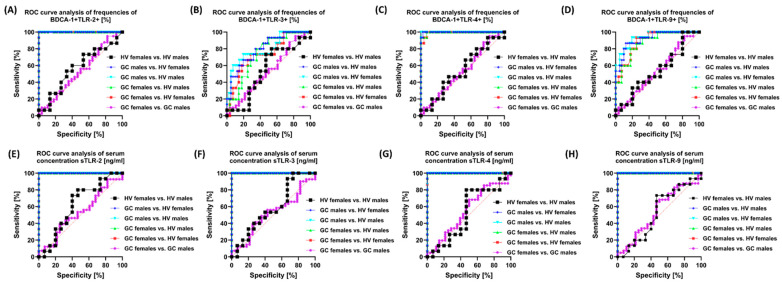
Graphical representation of selected ROC curves depending on the sex of recruited patients. (**A**–**D**) ROC curves presenting a comparison of sensitivity and specificity of the tested TLRs on BDCA-1; (**E**–**H**) ROC curves presenting a comparison of sensitivity and specificity of the tested concentrations of soluble forms of TLRs in the serum of patients.

In the case of analyses of the percentage of the studied TLRs on individual subpopulations of immune cells depending on the stage of the disease, we noted relatively high sensitivity and specificity of TLR-2, TLR-3, TLR-4, and TLR-9 between stage I and III; stage I and IV; stage II and IV; and stage II and III on BDCA-1 (Figure 9A–D) and BDCA-2, as well as classical and non-classical monocyte subpopulations. However, the highest parameters were observed for analyses of serum concentrations of individual soluble forms of the studied TLRs (Figure 9E–H). Similar trends were observed in the analyses of changes occurring in individual sexes of the recruited patients (Supplementary Materials Table S7 and Figure 10). Despite the lack of differences between women and men with GC and women and men from the group of healthy volunteers, the highest sensitivity and specificity in the remaining combinations were achieved for the parameters TLR-2 and TLR-4 present on BDCA-1 (Figure 10A–D) and BDCA-2, as well as all monocyte subpopulations (Supplementary Materials Table S7). Here, too, the highest parameters were observed for the analyses of serum concentrations of individual soluble forms of the studied TLRs (Figure 10E–H).

**Figure 11:**
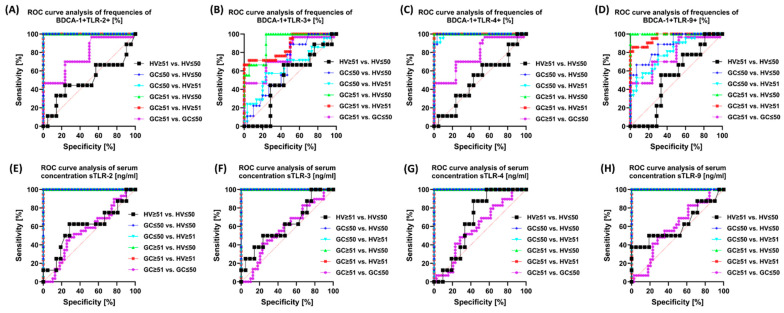
Graphical representation of selected ROC curves depending on the age of recruited patients. (**A**–**D**) ROC curves presenting a comparison of sensitivity and specificity of the tested TLRs on BDCA-1; (**E**–**H**) ROC curves presenting a comparison of sensitivity and specificity of the tested concentrations of soluble forms of TLRs in the serum of patients.

In the last step, we decided to analyze the differences in the age of the recruited patients (Supplementary Materials Table S8 and Figure 11). Similar to the gender of the patients, the observed trends were also preserved: the highest values were observed for serum concentration between the individual age groups and the percentage of TLR-2 and TLR-4 on individual immune cells, except differences between women and men with GC and women and men from the group of healthy volunteers (Figure 11).

## 4. Discussion

In the conducted study, we analyzed the importance of selected TLRs and a subpopulation of immune cells in patients with GC. The results indicate significant changes in the immune profiles of these patients compared to healthy volunteers, which may be important for understanding the pathogenesis of GC and the potential use of TLRs as prognostic biomarkers. Our study results indicated a significantly higher percentage of DCs and monocytes expressing TLR-2, TLR-3, TLR-4, and TLR-9 in patients with GC compared to the control group. An increase in the expression of these receptors was particularly noticeable with disease progression, suggesting their involvement in tumor progression. Higher expression of TLRs may promote the creation of an immunosuppressive environment supporting angiogenesis and invasion of tumor cells, according to previous reports [4,5,6,7]. However, it should be noted that these receptors are commonly activated in inflammatory conditions, which is typical for the tumor microenvironment. Therefore, it is difficult to clearly state whether the observed changes in TLR expression directly drive the development of GC or are only a secondary effect of chronic inflammation. The increase in TLR expression may be more of a marker of the immune reaction occurring in the body than a direct promoter of the neoplastic process. However, the patients recruited for the study were newly diagnosed and had not undergone any treatment before entering our study, which allows us to assume that the observed changes in TLR expression reflect natural processes occurring in the tumor microenvironment and are not the result of therapeutic interventions or response to treatment, which increases the reliability of the assessment of their role in the pathogenesis of GC.

According to the results, no significant differences in TLR expression were observed between patients with intestinal type and diffuse gastric cancer (GC), except for the levels of soluble forms of TLR-2, TLR-4, and TLR-9. This is unexpected, considering the significant differences in the biology of both histological types. Intestinal and diffuse types differ in etiology and pathogenesis, which could suggest more pronounced differences in TLR expression on monocyte and DC subpopulations. Our previous studies have shown such differences in the percentage of these receptors on T and B lymphocytes [45], which further emphasizes the complexity of the molecular mechanisms involved in GC. The lack of significant differences may be because the mechanisms of the immune response in these two types of cancer are more complicated and multifactorial than can be explained solely based on TLR expression. Additionally, other molecular factors, such as microenvironmental signals or cell interactions, may play a key role in regulating the immune response, indicating the need for further research to better understand these processes.

Analysis by patient age showed that older patients (≥51 years) had a higher percentage of monocytes and higher TLR expression on DCs and classical and non-classical monocytes. This may be due to the accumulation of environmental factors and age-related immunological changes. However, no significant differences were observed between the sexes, which suggests that sex does not significantly affect the analyzed immunological parameters in GC. Increased concentrations of soluble forms of TLR-2, TLR-3, TLR-4, and TLR-9 in the serum of GC patients indicate their potential importance as diagnostic biomarkers. The high sensitivity and specificity of these markers in distinguishing disease stages and analyses by sex emphasize their prognostic value. Despite the use of detailed patient inclusion and exclusion criteria, it is not entirely clear whether these soluble forms of TLRs reflect local activation of receptors in the tumor or are the result of a systemic inflammatory reaction. Additionally, many factors, such as infections, other inflammatory diseases, or even differences in metabolic status, could affect the levels of soluble forms of TLRs, potentially limiting their specificity for GC. Therefore, the use of these markers would require further studies to determine their specificity and sensitivity in distinguishing GC from other disease states.

The analyses performed in our study concern only one-time points (at the time of patient recruitment), and although they show some pictures of the state of newly diagnosed and untreated patients, they do not fully reflect the dynamics of changes in TLR expression and interactions between immune cells and tumors over time, which limits the possibility of inferring the long-term effects of these phenomena on disease progression. Nevertheless, they are an important step in understanding the mechanisms of GC, and their results emphasize the need for further long-term studies that could better capture these processes and their impact on disease progression. In our study, we aimed to identify TLRs on monocytes and DCs as potentially non-invasive prognostic markers based on measurements of their soluble forms in serum, which could be an alternative to more invasive diagnostic methods. There are numerous studies using tumor tissues in the literature that confirm the role of TLRs in the pathogenesis of GC, which additionally supports our hypothesis that TLRs may be important elements in the regulation of the immune response and progression of this tumor. Expression of TLRs on cancer cells can promote tumor growth by creating an environment conducive to immunosuppression, angiogenesis, and tumor invasion. In contrast, in immunocompetent cells such as monocytes and dendritic cells, TLRs serve a different function—they play a key role in initiating the immune response through antigen presentation. These different mechanisms of action of TLRs underscore their complex role in both promoting and combating tumor progression, depending on the cell type on which they are expressed.

Eskuri et al., in a study of tumor samples from 564 patients undergoing gastric cancer resection, used tissue microarrays to analyze TLR expression. The authors observed that an increased number of tumor cells expressing TLR-2 correlated with a longer five year survival of the patients, suggesting that TLR-2 may play a role as a favorable prognostic factor in gastric cancer [41]. In another study, conducted on gastric cancer samples from Iranian patients, an upregulation of the TLR-4 signaling pathway was observed. In particular, TLR-4 (*p* = 0.019), NF-κB (*p* = 0.047), IL-1β (*p* = 0.0096), and TNF-α (*p* = 0.048) were significantly increased in tumor tissues at different stages of gastric cancer, suggesting that TLR-4 and its associated proinflammatory cytokines may play a key role in tumor initiation and progression [32]. Further studies conducted by Oliveira et al. (2012) in Brazil focused on the polymorphisms of TLR2 and TLR4 genes and their association with the risk of gastric cancer. They showed that these polymorphisms, especially homozygous TLR-4 variants, were strongly associated with an increased risk of developing GC, further supporting the hypothesis of a key role of TLR-4 in the pathogenesis of gastric cancer [43].

Similar observations regarding intracellular TLRs come from the study of Eskuri et al. In this study, samples from 564 patients with gastric adenocarcinoma were reanalyzed, and it was shown that patients with high nuclear TLR-3 expression had significantly worse 5-year survival compared to patients with low expression of this receptor (crude hazard ratio 1.31, 95% CI 1.07–1.60). These results suggest that high nuclear TLR-3 expression may act as a negative prognostic factor in GC [42]. Fernandez-Garcia et al. also confirmed these observations using immunohistochemical analysis of tumor samples from 106 patients. They showed that high expression of TLR-3, TLR-4, and TLR-9 was significantly associated with shorter survival, especially in cases with lymph node involvement, further supporting their negative prognostic role [46]. Further evidence for TLRs’ role in GC pathogenesis comes from studies on TLR-9 expression. In the gp130 F/FH mouse model and patient samples, high TLR-9 expression was shown to promote the development of early stages of GC, supporting inflammatory processes and hyperplasia induced by *H. pylori* 39. Additional confirmation of the importance of TLR receptors in gastrointestinal cancers was provided by the studies of Diakowska et al., who analyzed the expression of TLR-2, TLR-4, and TLR-7 in patients with esophageal and gastroesophageal junction cancer. It was shown that the expression of TLR-2, TLR-4, and TLR-7 mRNA and their protein levels were significantly higher in cancer tissues compared to control tissues (*p* < 0.05) [47]. Moreover, patients with esophageal cancer and lymph node metastases had higher TLR-4 levels in tumor tissues, and in the subgroup with esophageal-gastric junction adenocarcinoma, significantly higher expression of TLR-7 mRNA and TLR-4, TLR-7, and TLR-9 proteins was observed [47]. The authors suggest that circulating TLR-4 proteins may act as potential markers of esophageal junction cancer. In connection with the above, contemporary studies indicate that TLRs play an essential role in the development of gastric cancer, both as potential promoters and inhibitors of neoplastic processes. As a result of the influence of these receptors on the tumor microenvironment by activating various immune cells, such as T lymphocytes, M1 macrophages, and NK cells, while reducing the number of suppressor cells (M2 macrophages, Treg), they can support the anti-tumor response. Therefore, these molecules could be helpful biomarkers for assessing the response to immunotherapies, such as checkpoint inhibitors (e.g., PD-1, PD-L1) [27].

### 4.1. Study Limitations

Despite the significant and interesting results obtained in our study, several limitations may affect their interpretation and generalizability to a wider population. First of all, the study was conducted on a relatively small sample size, including only 69 patients with gastric cancer (GC) and 30 control subjects. Such a number of participants may not reflect the full heterogeneity of the population, which significantly limits the generalizability of the results. Therefore, there is a need to conduct studies on larger, more diverse patient samples to obtain more representative data, especially in the context of the demographic and clinical diversity of patients.

Another significant limitation is the fact that the study was conducted in a single center, which additionally reduces the representativeness of the results and limits their relevance to the general population. Therefore, multicenter studies would be necessary to verify the obtained results in different populations and clinical settings. This would positively affect the reliability and consistency of the observations, especially about the heterogeneity of TLR expression in different patient populations.

In addition, the cross-sectional nature of the study does not allow for drawing clear conclusions regarding the causal relationship between TLR expression on monocytes and dendritic cells and the progression of gastric cancer. Longitudinal studies would be necessary to obtain more precise data regarding the temporal and causal relationship, which would allow changes in TLR expression over time and their impact on the clinical outcomes of patients.

Another limitation is that the study focused on measuring TLR expression levels, and the analysis of immune cell subpopulations was performed without including functional tests assessing the activity of TLR receptors and their related signaling pathways. Functional studies are necessary to better understand the role of TLRs in the pathogenesis of gastric cancer and their potential impact on tumor development and the immune response of patients.

Furthermore, the measurement of soluble TLRs (sTLRs) using enzyme-linked immunosorbent assays provided valuable information on systemic immune responses, but the interpretation of these results may be complicated by several additional factors that were not fully considered, such as the presence of other soluble immune factors. Therefore, future studies should include a more comprehensive analysis of the immune system, taking into account possible confounding factors such as differences in patient treatment, comorbidities, and other clinical variables that may influence TLR expression and immune cell function.

### 4.2. Could TLRs Be Potential Biomarkers for GC? What Do We Know, and What Should We Investigate?

Biomarkers are constantly being sought in scientific research to better understand disease mechanisms, personalize therapies, and improve treatment outcomes. “Possible” biomarker molecules are those that have been identified as promising in preliminary studies but require further validation before they are widely used in the clinic. These molecules are specific indicators of physiological, pathological, or therapeutic response processes that can be objectively assessed and measured. Could TLRs play such a role in GC? Literature data indicate that increased expression of TLRs, particularly TLR2, TLR4, and TLR9, in tumor cells and the tumor microenvironment is associated with GC progression. Chronic gastritis, especially associated with *H. pylori* infection, is one of the key risk factors for GC development. TLR4, which recognizes lipopolysaccharide (LPS) present in the cell membrane of Gram-negative bacteria, is key in the immune response to *H. pylori* infection. This infection leads to chronic inflammation, which may accelerate carcinogenesis through the secretion of proinflammatory cytokines such as TNF-alpha, IL-1β, and IL-6, which in turn increase the risk of DNA damage and mutations, which promotes the transformation of cancer cells. Overexpression of TLR4 in gastric cancer is also associated with more aggressive tumor characteristics, including the ability to invade and metastasize. Activation of the TLR4/NF-κB pathway leads to increased production of cytokines, growth factors, and proteases that promote angiogenesis and resistance to apoptosis, allowing cancer cells to survive and grow. Furthermore, high levels of TLR4 expression are associated with poorer prognosis and shorter survival time in GC patients [28,39,46,47].

TLR expression also has a significant impact on patient response to treatment. TLR4, activated in gastric cancer cells, is associated with resistance to chemotherapy, including common drugs used to treat this cancer, such as cisplatin and 5-fluorouracil (5-FU). Activation of the TLR4/NF-κB pathway promotes cancer cell survival and also increases the expression of anti-apoptosis proteins, such as Bcl-2, which results in reduced efficacy of chemotherapy. Studies indicate that gastric cancer patients with high TLR4 expression may require more intensive or individualized treatment regimens to overcome resistance to chemotherapy. Therefore, TLR4 may be a useful predictive biomarker that helps predict the efficacy of therapy and identify patients who require alternative treatment strategies [28,40,48,49,50,51].

Literature data suggest that TLRs may also play an important role in the context of GC immunotherapy. Immunotherapy, which involves boosting the patient’s immune response to fight the tumor, is a promising treatment method [52,53,54]. TLR agonists, such as TLR7 and TLR9 agonists, are currently being studied for their ability to enhance antitumor responses by activating dendritic cells and T cells. Activation of these receptors can stimulate the body’s immune response against cancer cells, making them potential adjuncts to immunotherapy. In the treatment of GC, TLR agonists are being studied in both preclinical and clinical trials, particularly in the context of immunotherapy and cancer vaccines. Imiquimod is one of the most studied TLR7 agonists, approved by the FDA for the treatment of superficial skin cancers such as basal cell carcinoma. In the context of gastric cancer, imiquimod has been studied primarily as an adjuvant in cancer vaccines. In the study by Wang et al. [55], vaccines were successfully created by covalently linking a TLR7 agonist (T7) to the GC antigen MG7-Ag, resulting in two variants: T7-ML (linear tetraepitope) and T7-MB (branched tetraepitope). In vitro, the vaccines rapidly induced the production of cytokines such as TNF-α and IL-12 in mouse dendritic cells (BMDC). The T7-MB vaccine showed the strongest immunogenic effect and the strongest ability to stimulate cytotoxic T lymphocyte (CTL) responses and antibody-dependent cytotoxicity (ADCC) activity. Moreover, in in vivo studies in mice, immunization with the T7-MB vaccine showed the greatest efficacy in reducing tumor mass and stimulating CTL and ADCC. The T7-MB vaccine, combined with 5-fluorouracil (5-FU) chemotherapy, showed a synergistic effect, leading to tumor reduction, prolonged survival, and enhanced T-cell response. The combination therapy of T7-MB and 5-FU resulted in increased T-cell activation (including CD8+ and CD4+) and a reduction in the number of immunosuppressive MDSCs, which was not possible with the vaccines alone [55,56].

Results from other studies have shown that imiquimod inhibits the proliferation of gastric adenocarcinoma SGC-7901 cells in a dose- and time-dependent manner, as shown by the MTT assay. Imiquimod induced both autophagy and apoptosis in the cells, and co-administration of the autophagy inhibitor 3-MA reduced the toxicity of imiquimod. Blocking autophagy with 3-MA also inhibited imiquimod-induced apoptosis, suggesting that autophagy acts as a mechanism leading to apoptosis and cell death of SGC-7901 cells [57]. Another example is TLR agonists such as CpG-ODN (TLR9 agonist) or R848 (TLR7/8 agonist), which have shown significant therapeutic effects as adjuvants in cancer immunotherapy, enhancing anti-tumor immune responses in various types of cancers, including glioblastoma multiforme and hematological malignancies [39,46,47,58,59,60]. However, their efficacy as monotherapy has been variable, suggesting that they may be more effective in combination with other treatments, such as immune checkpoint inhibitors such as PD-L1 inhibitors. These combinations have been shown to increase immune cell infiltration into tumors, increasing the efficacy of checkpoint blockade therapies [60,61,62,63]. TLR agonists such as Bacillus Calmette-Guérin (BCG), which targets TLR2 and TLR4, were among the first approved ligands used in cancer treatment, particularly in bladder cancer. Studies have shown that the use of TLR agonists as adjuvants in combination with chemotherapy, radiotherapy, and immunotherapy—including cancer vaccines—can improve clinical outcomes. For example, clinical trials combining TLR3 and TLR7/8 agonists with cancer vaccines have led to disease stabilization and tumor regression in some patients [58,59,60,61,62,63]. On the other hand, some TLRs can have pro-tumor effects, promoting tumor growth by stimulating the production of immunosuppressive cytokines. For example, activation of TLR4 has been associated with increased proliferation of gastric cancer cells, particularly in the context of *H. pylori* infection. In such cases, TLR inhibitors such as OPN-301 (anti-TLR2) and other TLR7/9 inhibitors have shown the potential to inhibit tumor growth in preclinical models [63,64,65,66]. Furthermore, assessment of TLR expression in tumor tissues can help identify patients who are most likely to respond to immunomodulatory therapy. For example, high TLR9 expression is associated with the activation of dendritic cells, which in turn can stimulate an effective anti-tumor response [60,61,62,63].

TLR expression especially TLR4, and TLR9, may act as a prognostic biomarker in gastric cancer. High levels of these receptors are associated with poorer prognosis, higher tumor aggressiveness, a higher risk of metastasis, and shorter patient survival. Therefore, assessment of TLR expression in gastric tumors may provide prognostic information that may help in treatment planning and monitoring the course of the disease. In the study conducted by Shirafkan et al., it was shown that TLR1 expression was significantly decreased in GC compared to other studied patient groups (patients with erosions, peptic ulcer disease, and GC). At the same time, TLR1 was significantly higher in the group of patients with erosions compared to the control group and GC, but there were no significant differences between the PUD, control, and erosion groups. TLR3 expression decreased with disease progression, from erosion to peptic ulcer disease to GC (*p* < 0.001). TLR4 expression did not show significant differences between the studied groups, but the highest expression of this receptor was observed in GC. In turn, TLR7 was strongly expressed in erosion, but its expression decreased significantly with the progression of the disease to PUD and GC. TLR9 did not show significant differences between the groups, but the lowest expression was observed in GC [63]. Another example is the study by Kasurinen et al., in which the tissue expression of TLR1, TLR2, TLR4, TLR5, TLR7, and TLR9 was assessed as potential prognostic biomarkers in GC. Immunohistochemistry was used to analyze tissue samples from 313 patients operated on for gastric adenocarcinoma. High expression of each of the TLRs studied correlated with high expression of the other and with intestinal histology (*p* < 0.001 for all). High TLR5 expression was associated with better prognosis of patients, and 5-year survival in this group was 53.4% (95% CI 43.4–63.4) compared to 37.6% (95% CI 30.0–45.2) in patients with low TLR5 levels (*p* = 0.014). A particularly favorable prognosis was observed in patients with stage II disease (HR 0.33; 0.13–0.83; *p* = 0.019) and in patients with intestinal cancer (HR 0.58; 95% CI 0.34–0.98; *p* = 0.043). The results of this study indicate for the first time that high TLR5 expression may be a marker of better prognosis in patients with GC, especially in stage II disease and in the case of intestinal cancer [64]. In the future, assessment of TLR expression in gastric cancer may become a key tool for personalizing therapy. Understanding which TLRs are overexpressed in a given tumor may help to tailor therapy to the needs of the patient. Patients with overexpression of TLR4 may require more aggressive therapy or a combination of chemotherapy with immunotherapy, while patients with overexpression of TLR9 may be good candidates for immunomodulatory therapies.

### 4.3. Future Perspectives—What Should Be the Subject of Future Research?

Most of the literature data on the role of TLRs as potential biomarker molecules come from studies using tissue material, which is associated with the invasiveness of the procedures leading to obtaining the research material. Future studies on gastric cancer should focus on identifying new, non-invasive biomarkers that could improve early diagnosis, monitoring disease progression, and predicting response to treatment. In particular, studies based on the analysis of immunocompetent cells can provide key information on the immunological mechanisms accompanying gastric cancer, which is important for the development of personalized therapeutic strategies. One of the key directions of future research should be functional studies of immunocompetent cells, which will allow for a better understanding of the activity of TLR receptors and their role in the pathogenesis of GC. Analysis of TLR expression alone provides information on the presence of these receptors, but only functional studies can explain how TLR activation affects the patient’s immune response. These studies should include the assessment of the activation of signaling pathways, such as NF-κB or MAPK, and the production of proinflammatory cytokines after TLR stimulation on immune cells. This will allow for a better understanding of how TLR interaction with cancer cells modulates the immune response and what mechanisms may lead to immunosuppression in the tumor microenvironment [65,66,67].

In addition, single-cell analyses may revolutionize the approach to studying immune cells in GC (currently studied are tumor cells) [68,69]. This advanced technique allows for the assessment of gene expression and function of individual cells at the single-cell level, which allows for more precise identification of immunocompetent cell subpopulations and their differences in immune response. In the case of GC, the use of single-cell RNA-seq technology may provide valuable information on the heterogeneity of immune cells, their dynamics during the disease, and interactions with tumor cells. Single-cell analyses may also help to identify unique gene expression profiles in different immune cell subpopulations, which is particularly important for understanding the mechanisms of resistance to immunotherapy. This type of research may also contribute to the discovery of new therapeutic targets that could be effectively used in the treatment of patients with GC.

The development of advanced immune profiling methods, such as flow cytometry, RNA sequencing, or proteomic techniques, enables detailed analysis of subpopulations of immunocompetent cells, and currently such studies are also conducted on tissue material [70,71,72]. These studies can provide valuable information on characteristic TLR expression patterns on T, B, and NK lymphocytes in patients with GC. Immune profiling will allow for the identification of specific changes in the immune response, which may have prognostic value and indicate potential therapeutic targets. Liquid biopsy is one of the most promising tools in modern cancer diagnostics, enabling non-invasive monitoring of the patient’s condition and disease progression. In the context of gastric cancer, the use of liquid biopsy allows for the analysis of circulating tumor cells (CTCs), circulating free tumor DNA (cfDNA), and immunocompetent cells [73,74,75]. This enables the detection of significant molecular changes in real-time, which enables dynamic monitoring of the response to therapy and the identification of new prognostic and predictive biomarkers. The use of liquid biopsy in the context of immunocompetent cells (such as monocytes, T cells, B cells, NK cells, and dendritic cells) is particularly interesting, as it allows monitoring of the patient’s immune response without the need for invasive procedures such as tissue biopsies. These cells play a key role in modulating the immune response against cancer, and their activation can provide information about the patient’s immune status and the effectiveness of anticancer therapies, including immunotherapy. Analysis of the expression of TLRs and other surface markers on immunocompetent cells in liquid biopsy will allow for the assessment of how immune activity changes in response to GC development and the treatment applied.

Another key element of future liquid biopsy research is the role of microRNAs (miRNAs) in modulating the function of immunocompetent cells. miRNAs are short, noncoding RNAs that regulate gene expression at the posttranscriptional level, influencing a variety of cellular processes, including immune function. miRNAs can modulate the activity of key signaling pathways in immunocompetent cells, such as TLRs, and regulate the immune response to tumors. In the case of GC, miRNAs can play an important role in inhibiting or promoting the immune response. For example, some miRNAs can suppress the activity of T lymphocytes or NK cells, which promotes the creation of an immunosuppressive tumor microenvironment. In contrast, other miRNAs can increase the activity of dendritic cells or monocytes, supporting an antitumor response. Studies on miRNAs in immunocompetent cells will allow us to understand how these small RNA molecules affect anti-tumor immunity and what prognostic and therapeutic significance they have [76,77,78].

An example is miR-155, which is strongly involved in the regulation of the immune response by controlling the activation of macrophages and monocytes and regulating TLR pathways. It has been shown that miR-155 may play a key role in promoting inflammatory immune responses, which may affect not only tumor progression but also the immunotherapeutic response. Detection of specific miRNAs in the blood of GC patients may provide valuable information on the immune status and tumor progression. miRNAs may act as non-invasive biomarkers monitoring the response of patients to treatment and identifying the risk of disease relapse [79,80,81]. For example, miR-21 and miR-34a have already been identified as gastric cancer-related miRNAs that may modulate the immune response and be potential biomarkers. Their levels in liquid biopsy can be used to monitor the body’s response to treatment and predict the clinical outcome of patients [82,83,84,85]. Combining miRNA expression analysis with the assessment of TLR activity in immunocompetent cells using liquid biopsy may open new possibilities in the diagnosis and treatment of gastric cancer. These studies will enable more precise monitoring of the dynamics of the immune response, which in turn will allow for early detection of changes in the patient’s condition and adjustment of therapeutic strategies in real-time.

In our opinion, future research on GC should focus on identifying new, non-invasive biomarkers, especially based on the analysis of immunocompetent cells and their TLR expression. A key direction will be the use of liquid biopsy technology, which allows monitoring of the immune status of patients in real-time, combined with functional analysis of immune cells and assessment of the role of miRNAs. Functional studies and single-cell technology will allow for a deeper understanding of the immunological mechanisms that affect the response to treatment and tumor progression. In the future, these approaches may significantly contribute to the development of personalized therapeutic strategies, improving the treatment outcomes of patients with GC.

## 5. Conclusions

Our study showed a significantly higher expression of TLR-2, TLR-3, TLR-4, and TLR-9 on monocyte subpopulations (classical, non-classical, and intermediate) and DCs (BDCA-1 and BDCA-2) in patients with GC compared to healthy volunteers. The exception was the expression of TLR-3 on classical monocytes, which did not differ significantly between groups. Additionally, the concentrations of soluble forms of TLRs in the serum of patients with GC were higher, and their increase correlated with the stage of disease progression, indicating a potentially crucial role of TLRs in tumor progression and their possible use as non-invasive biomarkers.

Despite significant differences in TLR expression on monocyte subpopulations and DCs, we did not observe significant changes between histological types of GC (intestinal and diffuse), except for soluble forms of TLR-2, TLR-4, and TLR-9. This result, although unexpected from the perspective of biological differences between histological types, suggests that the role of TLRs expressed on monocytes and DCs in the pathogenesis of GC may be more related to general immunological mechanisms and disease progression rather than directly to histological type. This indicates the need for further analysis of the microenvironmental and molecular mechanisms regulating the immune response in the context of GC.

Furthermore, analysis of age differences revealed that older patients (≥51 years) showed a higher percentage of monocytes and higher TLR expression on monocytes and DCs compared to younger patients. These results may suggest specific age-related immunological mechanisms that may influence the course of the disease, although further studies are necessary to better understand these relationships. The lack of significant differences in TLR expression on monocyte and DC subpopulations between sexes suggests that in this respect, sex does not have a significant influence on immunological parameters in GC.

Despite the interesting results, the study had some limitations. The cross-sectional nature of the study prevents us from drawing any clear conclusions about causal relationships between TLR expression on monocyte and DC subpopulations and tumor progression. Furthermore, the limited number of participants and the single-center nature of the study may affect the generalizability of the results. It is also worth noting that the increased levels of soluble TLRs may be a result of the general inflammatory response rather than a specific marker of GC. Further studies, controlling for potential confounding factors, are necessary to more precisely assess the specificity of TLRs as noninvasive biomarkers in GC.

In conclusion, our study suggests that TLR expression on monocyte and DC subpopulations may play a key role in the pathogenesis and progression of gastric cancer. Increased TLR expression and elevated serum levels of their soluble forms correlate with disease progression, which highlights their potential as diagnostic and, we hope, prognostic biomarkers. Moreover, due to their noninvasive nature, TLRs may represent promising therapeutic targets, especially in the context of advanced stages of GC. However, to fully understand the molecular mechanisms involved in TLR action and to evaluate their clinical application, further functional and multicenter studies, including larger patient populations, and long-term monitoring are necessary.

## Figures and Tables

**Table 1:** Basic analysis of peripheral blood morphology and assessment of the percentage of individual subpopulations of DCs and monocytes in patients with GC and HV.

Parameters	GC Patients		Healthy Volunteers		*p*-Value
	Mean SD	Median (Range)	Mean SD	Median (Range)	
Age	60.28 ± 14.04	58.00 (39.00–86.00)	58.97 ± 12.65	58.50 (37.00–82.00)	0.669
White blood cells [10^3^/µL]	7.65 ± 2.08	7.63 (4.12–10.88)	5.90 ± 0.92	5.97 (4.13–7.81)	0.000 *
Neutrophils [10^3^/µL]	4.35 ± 1.06	4.13 (2.20–6.10)	4.91 ± 1.03	5.22 (2.77–6.10)	0.021 *
Monocytes [10^3^/µL]	0.47 ± 0.12	0.47 (0.10–0.75)	0.49 ± 0.11	0.52 (0.32–0.68)	0.398
Lymphocytes [10^3^/µL]	2.42 ± 0.56	2.43 (1.47–4.10)	2.25 ± 0.50	2.27 (1.47–3.14)	0.244
Platelets [10^3^/µL]	183.03 ± 50.69	182.00 (100.00–268.00)	263.93 ± 56.46	255.50 (190.00–358.00)	0.000 *
Hemoglobin [g/dL]	12.33 ± 1.56	12.12 (10.00–14.90)	16.13 ± 1.15	16.29 (14.32–17.97)	0.000 *
Red blood cells [10^6^/µL]	3.35 ± 0.85	3.27 (2.01–4.99)	4.03 ± 0.54	3.98 (3.14–4.97)	0.000 *
BDCA-1+ occurrence in peripheral blood [%]	0.07 ± 0.04	0.06 (0.01–0.23)	0.24 ± 0.13	0.17 (0.13–0.52)	0.000 *
BDCA-2+ occurrence in peripheral blood [%]	0.08 ± 0.05	0.07 (0.01–0.22)	0.13 ± 0.07	0.12 (0.04–0.33)	0.000 *
Classical monocytes in peripheral blood [%]	85.70 ± 6.39	86.50 (67.47–96.42)	84.16 ± 8.88	80.19 (64.77–96.84)	0.451
Intermediate monocytes in peripheral blood [%]	8.19 ± 3.93	7.66 (2.14–20.46)	3.51 ± 0.95	3.53 (1.54–5.82)	0.000 *
Non-classical monocytes in peripheral blood [%]	5.24 ± 2.89	5.04 (0.93–15.40)	2.45 ± 0.96	2.52 (0.93–4.28)	0.000 *

The symbol * indicates statistically significant results.

**Table 2:** Evaluation of differences in the percentage of TLR-2, TLR-3, TLR-4, and TLR-9 expression on DCs and monocytes and the concentration of soluble forms in serum between GC patients and healthy volunteers.

Parameters		GC Patients		Healthy Volunteers		*p*-Value
		Mean SD	Median (Range)	Mean SD	Median (Range)	
Evaluation of the occurrence of the studied TLRs on DCs [%]	BDCA-1+ TLR-2+	19.54 ± 9.77	17.78 (3.19–48.00)	1.41 ± 0.71	1.27 (0.28–2.69)	0.000 *
	BDCA-1+ TLR-3+	5.06 ± 3.37	4.09 (0.81–14.47)	2.34 ± 1.48	1.74 (0.73–6.06)	0.000 *
	BDCA-1+ TLR-4+	14.56 ± 7.34	13.40 (3.06–30.77)	1.79 ± 0.93	1.66 (0.62–4.72)	0.000 *
	BDCA-1+ TLR-9+	8.34 ± 5.84	6.78 (1.59–28.07)	2.28 ± 1.55	2.15 (0.32–6.61)	0.000 *
	BDCA-2+ TLR-2+	12.26 ± 7.72	11.45 (2.21–43.86)	1.30 ± 0.76	1.19 (0.34–3.51)	0.000 *
	BDCA-2+ TLR-3+	5.63 ± 4.27	4.76 (0.30–22.22)	2.25 ± 1.31	2.05 (0.60–6.08)	0.000 *
	BDCA-2+ TLR-4+	13.12 ± 8.61	10.78 (2.80–45.83)	0.99 ± 0.58	0.90 (0.26–2.67)	0.000 *
	BDCA-2+ TLR-9+	10.17 ± 5.68	8.42 (1.49–27.27)	1.71 ± 1.00	1.56 (0.45–4.62)	0.000 *
Evaluation of the occurrence of the tested TLRs on monocytes [%]	Classical monocytes TLR-2+	7.25 ± 4.77	5.46 (2.01–21.37)	1.21 ± 0.67	1.10 (0.13–2.92)	0.000 *
	Classical monocytes TLR-3+	1.59 ± 2.22	0.71 (0.09–10.22)	1.01 ± 0.67	1.01 (0.13–2.33)	0.919
	Classical monocytes TLR-4+	6.30 ± 3.24	6.06 (0.88–14.77)	1.34 ± 0.74	1.21 (0.14–3.21)	0.000 *
	Classical monocytes TLR-9+	3.91 ± 4.95	1.92 (0.07–21.25)	1.10 ± 0.61	1.00 (0.12–2.65)	0.004 *
	Intermediate monocytes TLR-2	8.74 ± 7.98	6.84 (0.99–45.59)	0.93 ± 0.51	0.78 (0.21–2.57)	0.000 *
	Intermediate monocytes TLR-3+	4.67 ± 3.40	3.52 (0.38–15.98)	1.31 ± 0.72	1.11 (0.29–3.64)	0.000 *
	Intermediate monocytes TLR-4+	8.99 ± 8.18	5.96 (1.50–45.65)	0.78 ± 0.43	0.66 (0.17–2.16)	0.000 *
	Intermediate monocytes TLR-9+	4.71 ± 4.07	3.45 (0.43–22.76)	1.10 ± 0.61	0.93 (0.25–3.06)	0.000 *
	Non-classical monocytes TLR-2+	10.45 ± 5.92	9.62 (1.77–27.19)	0.46 ± 0.25	0.39 (0.10–1.28)	0.000 *
	Non-classical monocytes TLR-3+	5.31 ± 5.19	2.93 (0.24–25.43)	0.65 ± 0.36	0.55 (0.15–1.81)	0.000 *
	Non-classical monocytes TLR-4+	11.09 ± 5.47	9.66 (1.29–28.44)	0.39 ± 0.21	0.33 (0.09–1.07)	0.000 *
	Non-classical monocytes TLR-9+	5.37 ± 3.98	4.24 (0.53–21.53)	0.55 ± 0.30	0.46 (0.12–1.52)	0.000 *
Serum concentration [ng/mL]	sTLR-2	20.38 ± 7.20	18.91 (9.39–35.58)	3.78 ± 0.83	4.01 (2.09–4.90)	0.000 *
	sTLR-3	14.23 ± 4.78	14.99 (6.85–22.77)	1.96 ± 0.59	1.84 (1.01–2.98)	0.000 *
	sTLR-4	20.46 ± 5.94	21.43 (10.09–29.89)	3.15 ± 0.60	3.16 (2.04–3.95)	0.000 *
	sTLR-9	17.60 ± 5.67	18.46 (7.37–26.30)	1.88 ± 0.49	1.95 (1.02–2.96)	0.000 *

The symbol * indicates statistically significant results.

**Table 3:** Assessment of differences in the percentage of TLR-2, TLR-3, TLR-4, and TLR-9 expression on DCs and monocytes and the concentration of soluble forms in serum between GC patients depending on the age of the patients.

Parameters		GC ≥ 51 Years (Group 1)	GC ≤ 50 Years (Group 2)	HV ≤ 50 Years (Group 3)	HV ≥ 51 Years (Group 4)	*p*-Value					
		Median (Range)	Median (Range)	Median (Range)	Median (Range)	1 vs. 2	1 vs. 3	1 vs. 4	2 vs. 3	2 vs. 4	3 vs. 4
Evaluation of the occurrence of the studied TLRs on DCs [%]	BDCA-1+ TLR-2+	21.07 (10.74–48.00)	13.84 (3.19–40.63)	1.29 (0.33–2.69)	1.25 (0.28–2.69)	0.000 *	0.000 *	0.000 *	0.000 *	0.000 *	0.965
	BDCA-1+ TLR-3+	5.06 (2.38–14.47)	3.10 (0.81–14.04)	2.00 (0.87–3.57)	1.65 (0.73–6.06)	0.000 *	0.000 *	0.000 *	0.169	0.106	0.965
	BDCA-1+ TLR-4+	16.53 (8.16–30.77)	10.34 (3.06–30.68)	1.60 (0.74–3.26)	1.66 (0.62–4.72)	0.000 *	0.000 *	0.000 *	0.000 *	0.000 *	0.929
	BDCA-1+ TLR-9+	8.09 (3.85–28.07)	4.93 (1.59–24.44)	1.23 (1.00–3.84)	2.19 (0.32–6.61)	0.000 *	0.000 *	0.000 *	0.000 *	0.000 *	0.824
	BDCA-2+ TLR-2+	13.40 (5.77–43.86)	8.20 (2.21–37.78)	1.22 (0.41–2.84)	1.17 (0.34–3.51)	0.000 *	0.000 *	0.000 *	0.000 *	0.000 *	0.397
	BDCA-2+ TLR-3+	5.63 (2.46–22.22)	3.16 (0.30–19.61)	2.11 (0.71–4.92)	2.03 (0.60–6.08)	0.000 *	0.000 *	0.000 *	0.366	0.070	0.397
	BDCA-2+ TLR-4+	13.92 (6.88–45.83)	8.31 (2.80–39.60)	0.93 (0.31–2.16)	0.89 (0.26–2.67)	0.000 *	0.000 *	0.000 *	0.000 *	0.000 *	0.397
	BDCA-2+ TLR-9+	11.29 (5.33–27.27)	6.98 (1.49–23.39)	1.60 (0.54–3.74)	1.54 (0.45–4.62)	0.000 *	0.000 *	0.000 *	0.000 *	0.000 *	0.397
Evaluation of the occurrence of the tested TLRs on monocytes [%]	Classical monocytes TLR-2+	7.32 (3.36–20.39)	4.33 (2.01–21.37)	0.96 (0.46–2.27)	1.14 (0.13–2.92)	0.000 *	0.000 *	0.000 *	0.000 *	0.000 *	0.722
	Classical monocytes TLR-3+	1.12 (0.32–10.22)	0.50 (0.09–9.68)	1.25 (0.16–2.33)	0.76 (0.13–1.96)	0.000 *	0.585	0.089	0.093	0.047 *	0.397
	Classical monocytes TLR-4+	6.99 (3.35–14.77)	4.19 (0.88–14.01)	1.05 (0.51–2.49)	1.26 (0.14–3.21)	0.000 *	0.000 *	0.000 *	0.000 *	0.000 *	0.722
	Classical monocytes TLR-9+	2.87 (0.63–21.25)	1.23 (0.07–19.47)	0.87 (0.42–2.06)	1.04 (0.12–2.65)	0.000 *	0.002 *	0.000 *	0.909	0.887	0.722
	Intermediate monocytes TLR-2	6.38 (0.99–39.13)	7.53 (2.15–45.59)	0.78 (0.21–1.45)	0.85 (0.25–2.57)	0.266	0.000 *	0.000 *	0.000 *	0.000 *	0.722
	Intermediate monocytes TLR-3+	3.42 (0.63–13.41)	3.86 (0.38–15.98)	1.11 (0.29–2.05)	1.21 (0.35–3.64)	0.511	0.000 *	0.000 *	0.000 *	0.000 *	0.722
	Intermediate monocytes TLR-4+	5.61 (1.50–45.65)	6.61 (1.56–23.15)	0.66 (0.17–1.22)	0.72 (0.21–2.16)	0.457	0.000 *	0.000 *	0.000 *	0.000 *	0.722
	Intermediate monocytes TLR-9+	3.42 (0.51–15.84)	3.54 (0.43–22.76)	0.93 (0.25–1.72)	1.02 (0.29–3.06)	0.764	0.000 *	0.000 *	0.000 *	0.000 *	0.722
	Non-classical monocytes TLR-2+	11.03 (5.40–27.19)	7.17 (1.77–25.66)	0.39 (0.10–0.72)	0.43 (0.12–1.28)	0.000 *	0.000 *	0.000 *	0.000 *	0.000 *	0.722
	Non-classical monocytes TLR-3+	4.64 (1.30–20.77)	2.15 (0.24–25.43)	0.55 (0.15–1.02)	0.60 (0.17–1.81)	0.000 *	0.000 *	0.000 *	0.000 *	0.000 *	0.722
	Non-classical monocytes TLR-4+	11.84 (7.03–28.44)	8.16 (1.29–20.33)	0.33 (0.09–0.60)	0.36 (0.10–1.07)	0.000 *	0.000 *	0.000 *	0.000 *	0.000 *	0.722
	Non-classical monocytes TLR-9+	5.66 (1.90–17.02)	3.11 (0.53–21.53)	0.46 (0.12–0.86)	0.51 (0.15–1.52)	0.000 *	0.000 *	0.000 *	0.000 *	0.000 *	0.722
Serum concentration [ng/mL]	sTLR-2	18.21 (10.05–34.11)	19.03 (9.39–35.58)	3.58 (2.09–4.70)	4.08 (2.15–4.90)	0.430	0.000 *	0.000 *	0.000 *	0.000 *	0.429
	sTLR-3	11.59 (7.34–21.83)	15.69 (6.85–22.77)	2.08 (1.31–2.98)	1.83 (1.01–2.90)	0.330	0.000 *	0.000 *	0.000 *	0.000 *	0.324
	sTLR-4	19.45 (11.01–27.93)	23.28 (10.58–29.89)	2.93 (2.10–3.60)	3.24 (2.04–3.95)	0.226	0.000 *	0.000 *	0.000 *	0.000 *	0.184
	sTLR-9	17.70 (8.04–24.58)	20.02 (7.72–26.30)	1.97 (1.08–2.61)	1.92 (1.02–2.44)	0.221	0.000 *	0.000 *	0.000 *	0.000 *	0.324

The symbol * indicates statistically significant results.

## Data Availability

All data are available on written request from the first author of this publication.

## Supplementary Materials

The following supporting information can be downloaded at: https://www.mdpi.com/article/10.3390/cells13201708/s1. Table S1: Comparison of the obtained test results in assessing changes in morphological and immunological parameters between patients with diffuse and intestinal type and healthy volunteers; Table S2: Comparison of the obtained test results in terms of assessing changes in morphological and immunological parameters between patients in different stages of the disease; Table S3: Evaluation of differences in the percentage of TLR-2, TLR-3, TLR-4, and TLR-9 on dendritic cells and monocytes and the concentration of soluble forms in serum between GC patients depending on gender; Table S4: Evaluation of differences in the percentage of TLR-2, TLR-3, TLR-4, and TLR-9 on dendritic cells and monocytes and the concentration of soluble forms in serum between GC patients depending on age; Table S5: Spearman rank correlation analysis for GC patients; Table S6: ROC curve analysis for disease stage of GC patients; Table S7: ROC curve analysis for gender of GC and HV patients; Table S8: ROC curve analysis for age of GC and HV patients. Figure S1: Example Analysis of Monocyte Subpopulations in Peripheral Blood.
