# Supplementary material for: Immunological Strategies in Gastric Cancer: How Toll-like Receptors 2, -3, -4, and -9 on Monocytes and Dendritic Cells Depend on Patient Factors?
Source: Cells. 2024 Oct 16;13(20):1708. doi: 10.3390/cells13201708 (PMC11506270; doi:10.3390/cells13201708)
Supplement: Supplementary file 1 [file cells-13-01708-s001.zip › cells-3220019-supplementary.pdf]

Supplementary Materials Table S1- Comparison of the obtained test results in assessing changes in morphological and immunological parameters between patients with diffuse and intestinal type and healthy volunteers.

Parameters	Diffuse type		Intestinal type		HV		p-Value		
	Mean SD	Median (Range)	Mean SD	Median (Range)	Mean SD	Median (Range)	D vs. I	D vs. C	I vs. C
Age	58.66±13.61	56.00 (40.00-86.00)	61.94±14.27	65.00 (39.00-84.00)	58.97±12.65	58.50 (37.00-82.00)	0.380	0.870	0.359
White blood cells [10^3/ μl]	7.81±2.26	7.54 (4.12-10.88)	7.48±1.87	7.70 (4.19-10.57)	5.90±0.92	5.97 (4.13-7.81)	0.380	0.002	0.000
Neutrophils [10^3/ μl]	4.23±1.07	3.95 (2.20-6.09)	4.47±1.03	4.57 (2.20-6.10)	4.91±1.03	5.22 (2.77-6.10)	0.361	0.022	0.081
Monocytes [10^3/ μl]	0.46±0.13	0.45 (0.10-0.70)	0.48±0.11	0.48 (0.22-0.75)	0.49±0.11	0.52 (0.32-0.68)	0.531	0.349	0.588
Lymphocytes [10^3/ μl]	2.45±0.61	2.61 (1.47-4.10)	2.39±0.51	2.34 (1.57-4.10)	2.25±0.50	2.27 (1.47-3.14)	0.427	0.225	0.418
Platelets [10^3/μl]	180.06±47.22	183.00 (101.00-268.00)	186.09±53.86	173.00 (100.00-267.00)	263.93±56.46	255.50 (190.00-358.00)	0.629	0.000*	0.000*
Hemoglobin [g/dl]	12.29±1.62	12.07 (10.00-14.74)	12.37±1.50	14.90	16.13±1.15	16.29 (14.32-17.97)	0.707	0.000*	0.000*
Red blood cells [10^6/μl]	3.27±0.85	3.09 (2.01-4.99)	3.43±0.85	3.38 (2.01-4.90)	4.03±0.54	3.98 (3.14-4.97)	0.434	0.000	0.008*
BDCA1+ occurrence in peripheral blood [%]	0.46±0.12	0.43 (0.28-0.70)	0.48±0.12	0.47 (0.28-0.69)	0.24±0.13	0.17 (0.13-0.52)	0.434	0.000*	0.000*
BDCA2+ occurrence in peripheral blood [%]	0.27±0.13	0.27 (0.10-0.54)	0.30±0.13	0.29 (0.09-0.53)	0.13±0.07	0.12 (0.04-0.33)	0.348	0.000*	0.000*
Classical monocytes in peripheral blood [%]	85.63±5.95	85.57 (71.53-96.42)	85.78±6.81	87.52 (67.47-95.55)	84.16±8.88	80.19 (64.77-96.84)	0.523	0.363	0.693
Intermediate monocytes in peripheral blood [%]	8.23±3.62	8.30 (2.14-16.64)	8.14±4.23	7.15 (2.17-20.46)	3.51±0.95	3.53 (1.54-5.82)	0.515	0.000*	0.000*
Non-classical monocytes in peripheral blood [%]	5.29±2.52	5.39 (0.93-11.60)	5.17±3.23	4.61 (1.02-15.40)	2.45±0.96	2.52 (0.93-4.28)	0.523	0.000*	0.000*
BDCA-1+TLR-2+[%]	19.82±9.05	18.60 (3.19-38.95)	19.26±10.45	16.00 (6.12-48.00)	1.41±0.71	1.27 (0.28-2.69)	0.531	0.000*	0.000*
BDCA-1+TLR-3+[%]	5.09±3.07	4.48 (0.81-12.64)	5.02±3.66	3.51 (1.06-14.47)	2.34±1.48	1.74 (0.73-6.06)	0.523	0.000*	0.000*
BDCA-1+TLR-4+[%]	14.80±6.99	14.09 (3.06-29.82)	14.31±7.68	11.37 (3.85-30.77)	1.79±0.93	1.66 (0.62-4.72)	0.523	0.000*	0.000*
BDCA-1+TLR-9+[%]	8.42±5.26	7.34 (1.59-23.91)	8.26±6.38	6.03 (1.89-28.07)	2.28±1.55	2.15 (0.32-6.61)	0.515	0.000*	0.000*
BDCA-2+TLR-2+[%]	12.19±6.16	11.82 (2.21-27.78)	12.33±9.05	9.67 (2.47-43.86)	1.30±0.76	1.19 (0.34-3.51)	0.531	0.000*	0.000*

BDCA-2+TLR-3+[%]	5.53±3.43	5.05 (0.30-13.68)	5.74±4.98	3.82 (0.48-22.22)	2.25±1.31	2.05 (0.60-6.08)	0.523	0.000*	0.000*
BDCA-2+TLR-4+[%]	13.08±7.68	11.39 (2.80-38.52)	13.16±9.47	9.17 (2.86-45.83)	0.99±0.58	0.90 (0.26-2.67)	0.523	0.000*	0.000*
BDCA-2+TLR-9+[%]	10.31±5.21	9.93 (1.49-19.23)	10.03±6.12	7.84 (1.50-27.27)	1.71±1.00	1.56 (0.45-4.62)	0.539	0.000*	0.000*
Classical monocytes TLR-2+[%]	7.39±4.32	6.04 (2.04-19.90)	7.12±5.18	7.12 5.18	1.21±0.67	1.10 (0.13-2.92)	0.413	0.000*	0.000*
Classical monocytes TLR-3+[%]	1.53±1.94	0.97 (0.10-9.48)	1.66±2.48	0.59 (0.09-10.22)	1.01±0.67	1.01 (0.13-2.33)	0.400	0.779	0.635
Classical monocytes TLR-4+[%]	6.38±2.95	6.54 (1.25-13.14)	6.22±3.51	5.51 (0.88-14.77)	1.34±0.74	1.21 (0.14-3.21)	0.515	0.000*	0.000*
Classical monocytes TLR-9+[%]	4.03±4.92	2.31 (0.15-21.25)	3.78±4.97	1.45 (0.07-19.47)	1.10±0.61	1.00 (0.12-2.65)	0.485	0.001	0.086
Intermediate monocytes TLR-2+[%]	8.87±8.44	6.04 (0.99-45.59)	8.61±7.47	7.16 (1.13-39.13)	0.93±0.51	0.78 (0.21-2.57)	0.948	0.000*	0.000*
Intermediate monocytes TLR-3+[%]	4.91±3.78	4.12 (0.38-15.98)	4.42±2.94	3.08 (0.51-12.70)	1.31±0.72	1.11 (0.29-3.64)	0.853	0.000*	0.000*
Intermediate monocytes TLR-4+[%]	8.90±7.88	5.96 (1.56-35.41)	9.07±8.48	5.98 (1.50-45.65)	0.78±0.43	0.66 (0.17-2.16)	0.986	0.000*	0.000*
Intermediate monocytes TLR-9+[%]	5.19±4.73	3.63 (0.43-22.76)	4.21±3.17	3.07 (0.51-14.25)	1.10±0.61	0.93 (0.25-3.06)	0.508	0.000*	0.000*
Non-classical monocytes TLR-2+[%]	10.89±5.74	10.40 (2.07-27.19)	10.00±6.08	8.34 (1.77-25.66)	0.46±0.25	0.39 (0.10-1.28)	0.290	0.000*	0.000*
Non-classical monocytes TLR-3+[%]	5.41±4.42	4.31 (0.52-19.58)	5.21±5.88	2.59 (0.24-25.43)	0.65±0.36	0.55 (0.15-1.81)	0.342	0.000*	0.000*
Non-classical monocytes TLR-4+[%]	11.40±5.53	10.66 (1.96-28.44)	10.76±5.39	8.74 (1.29-22.46)	0.39±0.21	0.33 (0.09-1.07)	0.413	0.000*	0.000*
Non-classical monocytes TLR-9+[%]	5.63±3.63	4.97 (0.69-17.02)	5.10±4.30	3.46 (0.53-21.53)	0.55±0.30	0.46 (0.12-1.52)	0.301	0.000*	0.000*
sTLR2	23.00±5.20	23.00 (16.30-35.58)	18.83±7.37	18.56 (9.39-35.27)	3.78±0.83	4.01 (2.09-4.90)	0.007	0.000*	0.000*
sTLR3	14.76±4.48	15.11 (7.03-22.77)	13.68±5.01	13.15 (6.85-22.57)	1.96±0.59	1.84 (1.01-2.98)	0.374	0.000*	0.000*
sTLR4	22.18±4.46	23.28 (13.25-28.82)	18.70±5.78	18.14 (10.09-29.89)	3.15±0.60	3.16 (2.04-3.95)	0.012	0.000*	0.000*
sTLR9	19.69±3.84	20.02 (12.12-25.36)	16.32±6.07	17.32 (7.37-26.30)	1.88±0.49	1.95 (1.02-2.96)	0.029	0.000*	0.000*

The symbol * indicates statistically significant results.

Supplementary Materials Table S2- Comparison of the obtained test results in terms of assessing changes in morphological and immunological parameters between patients in different stages of the disease

Parameters	GC patients				p-Value					
	Stage I	Stage II	Stage III	Stage IV	I vs. II	I vs. III	I vs. IV	II vs. III	II vs. IV	III vs. IV
	Median (Range)	Median (Range)	Median (Range)	Median (Range)						
Age	58.00 (44.00-77.00)	48.50 (39.00-86.00)	70.00 (41.00-82.00)	57.00 (45.00-86.00)	0.000*	0.000*	0.000*	0.000*	0.000*	0.000*
White blood cells [10^3/ µl]	5.69 (4.19-10.74)	7.47 (4.12-10.75)	7.76 (4.92-10.57)	8.41 (4.86-10.88)	0.119	0.153	0.001*	0.631	0.457	0.101
Neutrophils [10^3/ µl]	5.11 (3.72-6.10)	4.15 (2.20-6.09)	4.44 (2.62-5.91)	3.69 (2.20-5.97)	0.922	0.551	0.634	0.581	0.892	0.352
Monocytes [10^3/ µl]	0.46 (0.22-0.68)	0.45 (0.28-0.67)	0.51 (0.10-0.70)	0.47 (0.29-0.75)	0.711	0.737	0.083	0.958	0.323	0.256
Lymphocytes [10^3/ µl]	2.20 (1.74-3.14)	2.42 (1.50-4.10)	2.47 (1.57-3.07)	2.70 (1.47-4.10)	0.216	0.478	0.704	0.488	0.267	0.954
Platelets [10^3/µl]	173.00 (100.00-265.00)	154.50 (101.00-268.00)	196.00 (105.00-260.00)	183.00 (119.00-267.00)	0.470	0.044	0.874	0.276	0.241	0.045
Hemoglobin [g/dl]	12.57 (10.63-14.90)	11.99 (10.04-14.37)	10.91 (10.09-14.63)	12.77 (10.00-14.89)	0.423	0.823	0.590	0.606	0.797	0.685
Red blood cells [10^6/µl]	3.34 (2.15-4.75)	3.18 (2.01-4.34)	3.27 (2.02-4.90)	3.17 (2.01-4.99)	0.423	0.823	0.590	0.606	0.797	0.685
BDCA1+ occurrence in peripheral blood [%]	0.47 (0.30-0.67)	0.44 (0.28-0.61)	0.46 (0.28-0.69)	0.44 (0.28-0.70)	0.066	0.000*	0.000*	0.025*	0.008*	0.383
BDCA2+ occurrence in peripheral blood [%]	0.36 (0.23-0.51)	0.32 (0.09-0.47)	0.27 (0.10-0.53)	0.21 (0.10-0.54)	0.060	0.000*	0.000*	0.028*	0.008*	0.383
Classical monocytes in peripheral blood [%]	89.65 (86.31-96.42)	87.50 (78.97-95.06)	83.99 (74.96-94.73)	81.70 (67.47-94.35)	0.060	0.000*	0.000*	0.028*	0.008*	0.383
Intermediate monocytes in peripheral blood [%]	5.42 (2.14-7.93)	7.10 (2.18-13.11)	9.00 (3.08-16.03)	10.26 (3.46-20.46)	0.060	0.000*	0.000*	0.028*	0.008*	0.383
Non-classical monocytes in peripheral blood [%]	2.93 (0.93-5.19)	4.54 (1.33-8.03)	5.94 (1.73-9.49)	6.55 (1.93-15.40)	0.129	0.00*	0.000*	0.025*	0.007*	0.383
BDCA-1+TLR-2+[%]	11.27 (3.19-18.37)	15.84 (6.71-32.29)	22.73 (8.11-36.36)	25.36 (8.54-48.00)	0.060	0.000*	0.000*	0.031*	0.008*	0.383
BDCA-1+TLR-3+[%]	2.63 (0.81-4.150	3.47 (1.12-10.20)	5.36 (1.19-11.35)	6.22 (1.49-14.47)	0.054	0.000*	0.000*	0.025*	0.007*	0.383
BDCA-1+TLR-4+[%]	8.88 (3.06-13.59)	11.00 (4.55-24.53)	16.67 (5.00-27.78)	18.64 (6.00-30.77)	0.119	0.033*	0.000*	0.423	0.008*	0.450
BDCA-1+TLR-9+[%]	4.22 (1.59-7.14)	5.89 (2.27-14.81)	8.65 (2.38-20.37)	10.53 (2.50-28.07)	0.060	0.000*	0.000*	0.028*	0.008*	0.367
BDCA-2+TLR-2+[%]	6.31	9.43	14.29	15.73	0.060	0.000*	0.000*	0.025*	0.008*	0.383

	(2.21-11.64)	(2.70-19.49)	(3.57-23.08)	(4.00-43.86)						
BDCA-2+TLR-3+[%]	2.58 (0.30-4.88)	3.91 (0.82-10.64)	6.06 (0.94-13.01)	7.14 (1.41-22.22)	0.060	0.000*	0.000*	0.028*	0.007*	0.383
BDCA-2+TLR-4+[%]	7.67 (2.80-10.91)	8.87 (2.99-21.62)	15.38 (3.61-26.39)	16.67 (4.65-45.83)	0.060	0.000*	0.000*	0.028*	0.008*	0.383
BDCA-2+TLR-9+[%]	5.50 (1.49-9.68)	8.08 (1.67-17.65)	12.24 (2.44-18.40)	13.79 (3.66-27.27)	0.060	0.000*	0.000*	0.025*	0.008*	0.383
Classical monocytes TLR-2+[%]	3.65 (2.01-5.79)	5.09 (2.04-13.81)	8.15 (2.29-16.60)	9.29 (2.60-21.37)	0.060	0.000*	0.000*	0.028*	0.007*	0.383
Classical monocytes TLR-3+[%]	0.37 (0.09-0.82)	0.58 (0.10-3.70)	1.21 (0.17-5.66)	1.46 (0.22-10.22)	0.060	0.000*	0.000*	0.028*	0.008*	0.432
Classical monocytes TLR-4+[%]	3.56 (0.88-6.20)	5.30 (1.99-9.55)	7.25 (2.03-12.67)	7.64 (2.62-14.77)	0.520	0.331	0.446	0.986	0.147	0.101
Classical monocytes TLR-9+[%]	0.65 (0.07-2.13)	1.45 (0.15-8.65)	3.36 (0.23-17.62)	4.38 (0.26-21.25)	0.101	0.001*	0.000*	0.028*	0.008*	0.383
Intermediate monocytes TLR-2+[%]	6.62 (2.15-45.59)	7.28 (1.51-13.41)	7.80 (0.99-20.83)	5.83 (1.72-27.58)	0.770	0.189	0.427	0.204	0.554	0.367
Intermediate monocytes TLR-3+[%]	3.13 (1.39-12.74)	3.46 (0.51-12.02)	2.74 (0.63-10.47)	5.50 (0.38-15.98)	0.495	0.246	0.046	0.986	0.217	0.256
Intermediate monocytes TLR-4+[%]	9.27 (2.51-45.65)	6.56 (2.54-33.12)	5.47 (1.50-19.38)	5.89 (2.76-23.15)	0.202	0.003*	0.000*	0.028*	0.007*	0.383
Intermediate monocytes TLR-9+[%]	2.56 (0.43-9.44)	3.54 (0.51-22.76)	3.20 (0.78-15.84)	5.00 (0.83-14.25)	0.800	0.911	0.704	0.817	0.241	0.322
Non-classical monocytes TLR-2+[%]	6.17 (1.77-21.14)	8.04 (2.07-15.79)	11.58 (3.31-23.76)	11.97 (3.92-27.19)	0.093	0.001*	0.000*	0.028*	0.008*	0.432
Non-classical monocytes TLR-3+[%]	1.71 (0.24-8.57)	2.59 (0.52-10.43)	5.26 (0.72-14.14)	6.98 (0.76-25.43)	0.216	0.003*	0.001*	0.028*	0.008*	0.432
Non-classical monocytes TLR-4+[%]	7.32 (1.29-9.73)	8.72 (1.96-17.99)	12.11 (3.32-22.46)	14.18 (4.34-28.44)	0.000*	0.000*	0.000*	0.000*	0.000*	0.000*
Non-classical monocytes TLR-9+[%]	2.17 (0.53-10.86)	3.51 (0.69-9.85)	6.49 (1.13-11.43)	7.18 (1.31-21.53)	0.216	0.003*	0.001*	0.025*	0.008*	0.432
sTLR2	10.55 (9.39-13.83)	18.16 (15.03-19.00)	19.68 (17.14-24.88)	28.61 (23.00-35.58)	0.000*	0.000*	0.000*	0.000*	0.000*	0.000*
sTLR3	7.70 (6.85-10.10)	11.07 (9.17-11.59)	16.92 (14.74-21.40)	18.31 (14.72-22.77)	0.000*	0.000*	0.000*	0.000*	0.000*	0.000*
sTLR4	12.43 (10.09-14.77)	15.68 (13.32-19.85)	23.47 (19.66-26.84)	26.76 (23.23-29.89)	0.000*	0.000*	0.000*	0.000*	0.000*	0.000*
sTLR9	9.07 (7.37-10.78)	14.26 (12.12-18.06)	20.18 (16.91-23.08)	23.54 (20.44-26.30)	0.000*	0.000*	0.000*	0.000*	0.000*	0.000*

The symbol * indicates statistically significant results.

Supplementary Materials Table S3-Evaluation of differences in the percentage of TLR-2, TLR-3, TLR-4 and TLR-9 on dendritic cells and monocytes and the concentration of soluble forms in serum between GC patients depending on gender

Parameters	GC female (group 1)	GC men (group 2)	HV female (group 3)	HV men (group 4)	p-Value					
	Median (Range)	Median (Range)	Median (Range)	Median (Range)	1 vs. 2	1 vs. 3	1 vs. 4	2 vs. 3	2 vs. 4	3 vs. 4
Age	57.00 (40.00-86.00)	63.00 (39.00-86.00)	55.00 (37.00-82.00)	60.00 (40.00-82.00)	0.985	0.772	0.870	0.660	0.798	0.838
White blood cells [10^3/ µl]	8.77 (4.30-10.88)	7.12 (4.12-10.68)	5.91 (4.13-7.81)	6.02 (4.20-7.48)	0.040*	0.001*	0.000*	0.036*	0.024*	1.000
Neutrophils [10^3/ µl]	4.61 (2.20-6.10)	4.03 (2.71-6.09)	5.31 (2.77-6.09)	5.13 (2.98-6.10)	0.359	0.356	0.201	0.061	0.036*	0.683
Monocytes [10^3/ µl]	0.48 (0.28-0.70)	0.44 (0.10-0.75)	0.51 (0.32-0.67)	0.55 (0.33-0.68)	0.203	0.772	0.605	0.558	0.198	0.512
Lymphocytes [10^3/ µl]	2.44 (1.47-4.10)	2.43 (1.50-3.50)	2.07 (1.57-3.10)	2.29 (1.47-3.14)	0.899	0.343	0.537	0.239	0.634	0.713
Platelets [10^3/µl]	199.50 (101.00-268.00)	164.00 (100.00-267.00)	259.00 (190.00-354.00)	252.00 (203.00-358.00)	0.120	0.004*	0.001*	0.000*	0.000*	0.567
Hemoglobin [g/dl]	12.01 (10.00-14.84)	12.15 (10.04-14.90)	15.28 (14.32-17.97)	16.37 (15.03-17.97)	0.640	0.0000*	0.0000*	0.0000*	0.0000*	0.116
Red blood cells [10^6/µl]	3.28 (2.12-4.99)	3.25 (2.01-4.90)	4.14 (3.30-4.97)	3.85 (3.14-4.69)	0.597	0.005*	0.064	0.001*	0.023*	0.233
BDCA1+ occurrence in peripheral blood [%]	0.46 (0.30-0.70)	0.46 (0.28-0.69)	0.19 (0.13-0.51)	0.16 (0.13-0.52)	0.597	0.0000*	0.0000*	0.0000*	0.0000*	0.389
BDCA2+ occurrence in peripheral blood [%]	0.27 (0.10-0.54)	0.29 (0.09-0.53)	0.12 (0.05-0.28)	0.12 (0.04-0.33)	0.841	0.0000*	0.0000*	0.0000*	0.0000*	0.713
Classical monocytes in peripheral blood [%]	86.79 (68.53-96.42)	86.32 (67.47-95.06)	78.90 (76.87-96.84)	81.35 (64.77-93.93)	0.632	0.520	0.356	0.812	0.674	0.713
Intermediate monocytes in peripheral blood [%]	7.58 (2.14-16.66)	7.66 (2.18-20.46)	3.53 (1.83-4.69)	3.54 (1.54-5.82)	0.632	0.000*	0.000*	0.000*	0.000*	0.902
Non-classical monocytes in peripheral blood [%]	4.96 (0.93-13.62)	5.11 (1.33-15.40)	2.70 (0.93-4.28)	2.51 (1.03-3.87)	0.632	0.001*	0.003*	0.000*	0.000*	0.967
BDCA-1+TLR-2+[%]	17.39 (3.19-40.63)	17.95 (6.71-48.00)	1.29 (0.33-2.69)	1.09 (0.28-2.69)	0.632	0.0000*	0.0000*	0.0000*	0.0000*	0.512
BDCA-1+TLR-3+[%]	3.90 (0.81-14.04)	4.11 (1.12-14.47)	1.39 (0.87-5.15)	2.00 (0.73-6.06)	0.614	0.009*	0.008*	0.0000*	0.0000*	0.683
BDCA-1+TLR-4+[%]	12.78 (3.06-30.68)	13.51 (4.55-30.77)	1.65 (0.74-3.96)	1.66 (0.62-4.72)	0.640	0.0000*	0.0000*	0.0000*	0.0000*	0.713
BDCA-1+TLR-9+[%]	6.57 (1.59-24.44)	6.87 (2.27-28.07)	2.19 (0.32-5.55)	2.10 (0.46-6.61)	0.606	0.0000*	0.0000*	0.0000*	0.0000*	0.744

BDCA-2+TLR-2+[%]	10.85 (2.21-37.78)	11.64 (2.70-43.86)	1.18 (0.51-2.95)	1.21 (0.34-3.51)	0.632	0.000*	0.000*	0.000*	0.000*	0.870
BDCA-2+TLR-3+[%]	4.44 (0.30-19.61)	4.76 (0.82-22.22)	2.04 (0.89-5.11)	2.10 (0.60-6.08)	0.640	0.003*	0.010*	0.0000*	0.0000*	0.870
BDCA-2+TLR-4+[%]	10.21 (2.80-39.60)	10.85 (2.99-45.83)	0.90 (0.39-2.24)	0.92 (0.26-2.67)	0.632	0.000*	0.000*	0.000*	0.000*	0.870
BDCA-2+TLR-9+[%]	8.33 (1.49-23.39)	9.60 (1.67-27.27)	1.55 (0.68-3.88)	1.60 (0.45-4.62)	0.640	0.000*	0.000*	0.000*	0.000*	0.870
Classical monocytes TLR-2+[%]	5.38 (2.01-21.37)	5.50 (2.04-20.39)	1.28 (0.13-2.45)	0.82 (0.15-2.92)	0.766	0.0000*	0.0000*	0.0000*	0.0000*	0.098
Classical monocytes TLR-3+[%]	0.69 (0.09-9.68)	0.77 (0.10-10.22)	0.76 (0.16-1.75)	1.49 (0.13-2.33)	0.785	0.753	0.537	0.558	0.533	0.106
Classical monocytes TLR-4+[%]	5.96 (0.88-14.01)	6.06 (1.99-14.77)	1.40 (0.14-2.69)	0.90 (0.17-3.21)	0.632	0.0000*	0.0000*	0.0000*	0.0000*	0.098
Classical monocytes TLR-9+[%]	1.73 (0.07-19.47)	1.97 (0.15-21.25)	1.16 (0.12-2.23)	0.75 (0.14-2.65)	0.658	0.175	0.050*	0.064	0.009*	0.098
Intermediate monocytes TLR-2+[%]	6.00 (1.13-29.73)	7.61 (0.99-45.59)	0.85 (0.21-2.57)	0.71 (0.25-1.67)	0.378	0.0000*	0.0000*	0.0000*	0.0000*	0.250
Intermediate monocytes TLR-3+[%]	3.28 (0.38-12.74)	3.68 (0.51-15.98)	1.21 (0.29-3.64)	1.01 (0.35-2.36)	0.908	0.0000*	0.0000*	0.0000*	0.0000*	0.250
Intermediate monocytes TLR-4+[%]	5.93 (1.50-35.41)	6.03 (1.56-45.65)	0.72 (0.17-2.16)	0.60 (0.21-1.40)	0.899	0.0000*	0.0000*	0.0000*	0.0000*	0.250
Intermediate monocytes TLR-9+[%]	3.71 (0.43-22.76)	2.93 (0.51-15.84)	1.02 (0.25-3.06)	0.85 (0.29-1.98)	0.540	0.0000*	0.0000*	0.0000*	0.0000*	0.250
Non-classical monocytes TLR-2+[%]	9.72 (1.77-25.66)	9.62 (2.07-27.19)	0.43 (0.10-1.28)	0.35 (0.12-0.83)	0.985	0.0000*	0.0000*	0.0000*	0.0000*	0.250
Non-classical monocytes TLR-3+[%]	3.08 (0.24-25.43)	2.93 (0.52-20.77)	0.60 (0.15-1.81)	0.50 (0.17-1.17)	0.956	0.0000*	0.0000*	0.0000*	0.0000*	0.250
Non-classical monocytes TLR-4+[%]	9.38 (1.29-21.08)	9.68 (1.96-28.44)	0.36 (0.09-1.07)	0.30 (0.10-0.70)	0.693	0.0000*	0.0000*	0.0000*	0.0000*	0.250
Non-classical monocytes TLR-9+[%]	4.34 (0.53-21.53)	4.24 (0.69-17.02)	0.51 (0.12-1.52)	0.42 (0.15-0.99)	0.995	0.0000*	0.0000*	0.0000*	0.0000*	0.250
sTLR2	19.01 (9.63-34.37)	18.87 (9.39-35.58)	4.08 (2.27-4.80)	3.56 (2.09-4.90)	0.684	0.0000*	0.0000*	0.0000*	0.0000*	0.285
sTLR3	15.17 (7.03-22.00)	14.74 (6.85-22.77)	1.98 (1.26-2.90)	1.83 (1.01-2.98)	0.794	0.0000*	0.0000*	0.0000*	0.0000*	0.461
sTLR4	23.57 (11.01-28.82)	20.62 (10.09-29.89)	3.15 (2.08-3.88)	3.60 (2.04-3.95)	0.462	0.0000*	0.0000*	0.0000*	0.0000*	0.806
sTLR9	20.74 (8.04-25.36)	18.06 (7.37-26.30)	1.92 (1.08-2.96)	2.12 (1.02-2.61)	0.532	0.0000*	0.0000*	0.0000*	0.0000*	0.806

The symbol * indicates statistically significant results.

Supplementary Materials Table S4-Evaluation of differences in the percentage of TLR-2, TLR-3, TLR-4 and TLR-9 on dendritic cells and monocytes and the concentration of soluble forms in serum between GC patients depending on age

Parameters	GC under 50 (group 2)	GC above 50 (group 1)	HV under 50 (group 3)	HV above (group 4)	p-Value					
	**Median (Range)**	**Median (Range)**	**Median (Range)**	**Median (Range)**	**1 vs. 2**	**1 vs. 3**	**1 vs. 4**	**2 vs. 3**	**2 vs. 4**	**3 vs. 4**
Age	47.00 (39.00-50.00)	72.50 (54.00-86.00)	44.00 (37.00-50.00)	65.00 (53.00-82.00)	0.000*	0.000*	0.014*	0.114	0.000*	0.000*
White blood cells [10^3/ µl]	7.89 (4.12-10.63)	7.42 (4.19-10.88)	5.36 (4.61-7.81)	6.04 (4.13-7.48)	0.736	0.008*	0.001*	0.046*	0.021*	0.283
Neutrophils [10^3/ µl]	4.14 (2.20-6.10)	4.08 (2.62-5.87)	5.83 (2.98-6.10)	4.83 (2.77-5.99)	0.421	0.000*	0.095	0.074	0.769	0.032*
Monocytes [10^3/ µl]	0.52 (0.32-0.68)	0.44 (0.10-0.75)	0.58 (0.33-0.68)	0.46 (0.32-0.66)	0.017*	0.014*	0.446	0.241	0.219	0.063
Lymphocytes [10^3/ µl]	2.62 (1.47-4.10)	2.30 (1.64-3.50)	2.75 (1.70-3.14)	1.98 (1.47-3.06)	0.190	0.103	0.083	0.682	0.026*	0.019*
Platelets [10^3/µl]	171.50 (104.00-267.00)	188.50 (100.00-268.00)	221.00 (193.00-318.00)	259.00 (190.00-358.00)	0.552	0.005*	0.000*	0.005*	0.000*	0.226
Hemoglobin [g/dl]	12.22 (10.15-14.84)	12.11 (10.00-14.90)	16.22 (14.38-17.31)	16.35 (14.32-17.97)	0.717	0.000*	0.000*	0.000*	0.000*	0.965
Red blood cells [10^6/µl]	3.04 (2.01-4.90)	3.38 (2.01-4.99)	3.84 (3.30-4.97)	4.03 (3.14-4.96)	0.207	0.097	0.011*	0.019*	0.001*	0.824
BDCA1+ occurrence in peripheral blood [%]	0.43 (0.28-0.69)	0.47 (0.28-0.70)	0.17 (0.13-0.42)	0.19 (0.13-0.52)	0.207	0.000*	0.000*	0.000*	0.000*	0.283
BDCA2+ occurrence in peripheral blood [%]	0.29 (0.10-0.53)	0.22 (0.09-0.54)	0.11 (0.05-0.23)	0.12 (0.04-0.33)	0.113	0.002*	0.000*	0.000*	0.000*	0.929
Classical monocytes in peripheral blood [%]	88.28 (68.53-96.42)	84.40 (67.47-90.66)	91.96 (77.11-96.84)	78.87 (64.77-93.93)	0.000*	0.040*	0.771	0.909	0.003*	0.137
Intermediate monocytes in peripheral blood [%]	6.29 (2.14-16.66)	8.81 (4.90-20.46)	3.86 (1.83-5.82)	3.53 (1.54-4.69)	0.000*	0.000*	0.000*	0.064	0.001*	0.193
Non-classical monocytes in peripheral blood [%]	3.77 (0.93-13.62)	5.68 (2.50-15.40)	2.18 (1.18-3.02)	2.53 (0.93-4.28)	0.000*	0.000*	0.000*	0.093	0.070	0.476

The symbol * indicates statistically significant results.

Supplementary Materials Table S5- Spearman rank correlation analysis for GC patients

Pair of variables	R	t(N-2)	p-Value
Classical monocytes in peripheral blood [%] & Intermediate monocytes in peripheral blood [%]	-1.000	-1105.642	0.000*
Classical monocytes in peripheral blood [%] & Non-classical monocytes in peripheral blood [%]	-1.000	-1105.642	0.000*
Classical monocytes in peripheral blood [%] & BDCA-2+TLR-4+ [%]	-1.000	-957.503	0.000*
Classical monocytes in peripheral blood [%] & BDCA-2+TLR-2+ [%]	-1.000	-781.781	0.000*
Classical monocytes in peripheral blood [%] & BDCA-1+TLR-4+ [%]	-1.000	-723.778	0.000*
Classical monocytes in peripheral blood [%] & BDCA-2+TLR-3+ [%]	-1.000	-638.304	0.000*
Classical monocytes in peripheral blood [%] & BDCA-1+TLR-2+ [%]	-1.000	-638.303	0.000*
Classical monocytes in peripheral blood [%] & BDCA-1+TLR-3+ [%]	-1.000	-577.355	0.000*
Classical monocytes in peripheral blood [%] & Classical monocytes TLR-4+ [%]	-1.000	-531.088	0.000*
Classical monocytes in peripheral blood [%] & BDCA-1+TLR-9+ [%]	-1.000	-531.080	0.000*
Classical monocytes in peripheral blood [%] & BDCA-2+TLR-9+ [%]	-1.000	-494.399	0.000*
Classical monocytes in peripheral blood [%] & Classical monocytes TLR-9+ [%]	-0.999	-167.170	0.000*
Classical monocytes in peripheral blood [%] & Non-classical monocytes TLR-4+ [%]	-0.994	-72.507	0.000*
Classical monocytes in peripheral blood [%] & Classical monocytes TLR-2+ [%]	-0.990	-57.043	0.000*
Classical monocytes in peripheral blood [%] & Classical monocytes TLR-3+ [%]	-0.983	-43.702	0.000*
Classical monocytes in peripheral blood [%] & Non-classical monocytes TLR-3+ [%]	-0.946	-23.803	0.000*
Classical monocytes in peripheral blood [%] & Non-classical monocytes TLR-9+ [%]	-0.930	-20.640	0.000*
Classical monocytes in peripheral blood [%] & Non-classical monocytes TLR-2+ [%]	-0.928	-20.316	0.000*
BDCA-2+ occurrence in peripheral blood [%] & BDCA-1+TLR-9+ [%]	-0.666	-7.313	0.000*
BDCA-2+ occurrence in peripheral blood [%] & BDCA-1+TLR-3+ [%]	-0.666	-7.312	0.000*
BDCA-2+ occurrence in peripheral blood [%] & BDCA-1+TLR-4+ [%]	-0.666	-7.310	0.000*
BDCA-2+ occurrence in peripheral blood [%] & Classical monocytes TLR-9+ [%]	-0.666	-7.308	0.000*
BDCA-2+ occurrence in peripheral blood [%] & BDCA-2+TLR-9+ [%]	-0.666	-7.305	0.000*
BDCA-2+ occurrence in peripheral blood [%] & Non-classical monocytes in peripheral blood [%]	-0.665	-7.298	0.000*
BDCA-2+ occurrence in peripheral blood [%] & BDCA-2+TLR-3+ [%]	-0.665	-7.295	0.000*
BDCA-2+ occurrence in peripheral blood [%] & BDCA-1+TLR-2+ [%]	-0.665	-7.294	0.000*
BDCA-2+ occurrence in peripheral blood [%] & Intermediate monocytes in peripheral blood [%]	-0.665	-7.294	0.000*
BDCA-2+ occurrence in peripheral blood [%] & BDCA-2+TLR-4+ [%]	-0.665	-7.289	0.000*
BDCA-2+ occurrence in peripheral blood [%] & BDCA-2+TLR-2+ [%]	-0.664	-7.273	0.000*
BDCA-2+ occurrence in peripheral blood [%] & Classical monocytes TLR-4+ [%]	-0.664	-7.269	0.000*
BDCA-2+ occurrence in peripheral blood [%] & Non-classical monocytes TLR-4+ [%]	-0.652	-7.044	0.000*

BDCA-2+ occurrence in peripheral blood [%] & Classical monocytes TLR-2+ [%]	-0.650	-6.996	0.000*
BDCA-2+ occurrence in peripheral blood [%] & Classical monocytes TLR-3+ [%]	-0.644	-6.885	0.000*
BDCA-2+ occurrence in peripheral blood [%] & Non-classical monocytes TLR-3+ [%]	-0.611	-6.321	0.000*
BDCA-2+ occurrence in peripheral blood [%] & Non-classical monocytes TLR-9+ [%]	-0.599	-6.121	0.000*
BDCA-2+ occurrence in peripheral blood [%] & Non-classical monocytes TLR-2+ [%]	-0.596	-6.079	0.000*
BDCA-2+ occurrence in peripheral blood [%] & sTLR3	-0.567	-5.635	0.000*
Classical monocytes in peripheral blood [%] & sTLR3	-0.557	-5.491	0.000*
BDCA-2+ occurrence in peripheral blood [%] & sTLR2	-0.555	-5.455	0.000*
Stage TNM & Classical monocytes in peripheral blood [%]	-0.541	-5.271	0.000*
Classical monocytes in peripheral blood [%] & sTLR2	-0.537	-5.214	0.000*
Classical monocytes in peripheral blood [%] & sTLR9	-0.515	-4.924	0.000*
Classical monocytes in peripheral blood [%] & sTLR4	-0.513	-4.887	0.000*
Stage TNM & BDCA-2+ occurrence in peripheral blood [%]	-0.468	-4.329	0.000*
Neutrophils & BDCA-2+TLR-2+ [%]	-0.466	-4.306	0.000*
Neutrophils & Classical monocytes TLR-4+ [%]	-0.465	-4.296	0.000*
Neutrophils & BDCA-1+TLR-9+ [%]	-0.465	-4.294	0.000*
Neutrophils & BDCA-2+TLR-9+ [%]	-0.464	-4.292	0.000*
Neutrophils & BDCA-2+TLR-4+ [%]	-0.464	-4.291	0.000*
Neutrophils & BDCA-2+TLR-3+ [%]	-0.464	-4.289	0.000*
Neutrophils & BDCA-1+TLR-4+ [%]	-0.464	-4.288	0.000*
Neutrophils & BDCA-1+TLR-2+ [%]	-0.464	-4.287	0.000*
Neutrophils & Intermediate monocytes in peripheral blood [%]	-0.464	-4.284	0.000*
Neutrophils & BDCA-1+TLR-3+ [%]	-0.464	-4.283	0.000*
Neutrophils & Non-classical monocytes in peripheral blood [%]	-0.464	-4.283	0.000*
Neutrophils & Classical monocytes TLR-2+ [%]	-0.461	-4.257	0.000*
Neutrophils & Classical monocytes TLR-9+ [%]	-0.461	-4.255	0.000*
Neutrophils & Non-classical monocytes TLR-4+ [%]	-0.460	-4.246	0.000*
Neutrophils & Classical monocytes TLR-3+ [%]	-0.449	-4.113	0.000*
BDCA-2+ occurrence in peripheral blood [%] & sTLR9	-0.441	-4.025	0.000*
BDCA-2+ occurrence in peripheral blood [%] & sTLR4	-0.434	-3.946	0.000*
Neutrophils & Non-classical monocytes TLR-3+ [%]	-0.422	-3.805	0.000*
Neutrophils & Non-classical monocytes TLR-9+ [%]	-0.412	-3.698	0.000*
Neutrophils & Non-classical monocytes TLR-2+ [%]	-0.411	-3.689	0.000*

Age & Classical monocytes in peripheral blood [%]	-0.410	-3.685	0.000*
Stage TNM & Neutrophils	-0.333	-2.891	0.005*
Neutrophils & sTLR2	-0.310	-2.672	0.009*
Monocytes & Classical monocytes TLR-9+ [%]	-0.287	-2.449	0.017*
Monocytes & Non-classical monocytes TLR-4+ [%]	-0.282	-2.409	0.019*
Monocytes & BDCA-1+TLR-9+ [%]	-0.281	-2.395	0.019*
Monocytes & BDCA-2+TLR-9+ [%]	-0.279	-2.381	0.020*
Monocytes & BDCA-1+TLR-2+ [%]	-0.279	-2.380	0.020*
Monocytes & BDCA-2+TLR-4+ [%]	-0.279	-2.375	0.020*
Monocytes & BDCA-1+TLR-4+ [%]	-0.278	-2.371	0.021*
Monocytes & BDCA-1+TLR-3+ [%]	-0.278	-2.370	0.021*
Monocytes & Non-classical monocytes in peripheral blood [%]	-0.278	-2.367	0.021*
Monocytes & Classical monocytes TLR-4+ [%]	-0.278	-2.367	0.021*
Monocytes & Intermediate monocytes in peripheral blood [%]	-0.278	-2.365	0.021*
Monocytes & BDCA-2+TLR-3+ [%]	-0.277	-2.362	0.021*
Monocytes & BDCA-2+TLR-2+ [%]	-0.276	-2.351	0.022*
Monocytes & Classical monocytes TLR-2+ [%]	-0.273	-2.325	0.023*
Neutrophils & sTLR9	-0.268	-2.275	0.026*
Monocytes & Classical monocytes TLR-3+ [%]	-0.266	-2.263	0.027*
Monocytes & Non-classical monocytes TLR-9+ [%]	-0.254	-2.152	0.035*
Neutrophils & sTLR4	-0.253	-2.142	0.036*
Neutrophils & sTLR3	-0.252	-2.130	0.037*
Monocytes & Non-classical monocytes TLR-3+ [%]	-0.250	-2.116	0.038*
Monocytes & Non-classical monocytes TLR-2+ [%]	-0.250	-2.110	0.039*
Gender & White blood cells	-0.248	-2.097	0.040*
Age & Monocytes	-0.240	-2.027	0.047*
Age & Intermediate monocytes TLR-2 [%]	-0.238	-2.008	0.049*
Age & sTLR4	0.241	2.030	0.046*
Age & sTLR9	0.241	2.036	0.046*
Intermediate monocytes TLR-9+ [%] & Intermediate monocytes TLR-4+ [%]	0.244	2.062	0.043*
Stage TNM & Intermediate monocytes TLR-9+ [%]	0.253	2.136	0.036*
Lymphocytes & sTLR2	0.263	2.235	0.029*
Monocytes & Classical monocytes in peripheral blood [%]	0.278	2.368	0.021*

Lymphocytes & sTLR3	0.294	2.513	0.014*
Intermediate monocytes TLR-9+ [%] & sTLR3	0.310	2.671	0.009*
Neutrophils & Monocytes	0.318	2.742	0.008*
Intermediate monocytes TLR-9+ [%] & sTLR2	0.326	2.825	0.006*
Lymphocytes & Intermediate monocytes TLR-9+ [%]	0.361	3.165	0.002*
Age & Non-classical monocytes TLR-2+ [%]	0.380	3.359	0.001*
Age & Non-classical monocytes TLR-9+ [%]	0.381	3.370	0.001*
Red blood cells & BDCA-2+ occurrence in peripheral blood [%]	0.382	3.388	0.001*
BDCA-1+ occurrence in peripheral blood [%] & BDCA-2+ occurrence in peripheral blood [%]	0.382	3.388	0.001*
Age & Non-classical monocytes TLR-3+ [%]	0.386	3.429	0.001*
Age & Classical monocytes TLR-2+ [%]	0.402	3.590	0.001*
Age & Classical monocytes TLR-3+ [%]	0.405	3.624	0.001*
Age & BDCA-1+TLR-3+ [%]	0.408	3.660	0.000*
Age & BDCA-2+TLR-4+ [%]	0.409	3.670	0.000*
Age & BDCA-1+TLR-9+ [%]	0.409	3.670	0.000*
Age & BDCA-2+TLR-9+ [%]	0.409	3.672	0.000*
Age & Classical monocytes TLR-9+ [%]	0.410	3.675	0.000*
Age & BDCA-1+TLR-4+ [%]	0.410	3.675	0.000*
Age & BDCA-2+TLR-3+ [%]	0.410	3.676	0.000*
Age & BDCA-2+TLR-2+ [%]	0.410	3.677	0.000*
Age & BDCA-1+TLR-2+ [%]	0.410	3.682	0.000*
Age & Non-classical monocytes in peripheral blood [%]	0.410	3.682	0.000*
Age & Classical monocytes TLR-4+ [%]	0.411	3.687	0.000*
Age & Intermediate monocytes in peripheral blood [%]	0.411	3.688	0.000*
Age & Non-classical monocytes TLR-4+ [%]	0.414	3.724	0.000*
Monocytes & Lymphocytes	0.433	3.930	0.000*
Intermediate monocytes TLR-3+ [%] & Intermediate monocytes TLR-2 [%]	0.440	4.012	0.000*
Neutrophils & Classical monocytes in peripheral blood [%]	0.464	4.288	0.000*
Non-classical monocytes TLR-2+ [%] & sTLR9	0.467	4.323	0.000*
Non-classical monocytes TLR-2+ [%] & sTLR4	0.469	4.345	0.000*
Non-classical monocytes TLR-9+ [%] & sTLR4	0.470	4.354	0.000*
Non-classical monocytes TLR-2+ [%] & sTLR2	0.470	4.355	0.000*
Non-classical monocytes TLR-9+ [%] & sTLR2	0.472	4.382	0.000*

Stage TNM & Non-classical monocytes TLR-2+ [%]	0.477	4.446	0.000*
Non-classical monocytes TLR-3+ [%] & sTLR9	0.479	4.464	0.000*
Stage TNM & Non-classical monocytes TLR-9+ [%]	0.479	4.466	0.000*
sTLR4 & Non-classical monocytes TLR-3+ [%]	0.480	4.477	0.000*
Non-classical monocytes TLR-3+ [%] & sTLR2	0.488	4.577	0.000*
Non-classical monocytes TLR-2+ [%] & sTLR3	0.494	4.648	0.000*
Non-classical monocytes TLR-9+ [%] & sTLR3	0.496	4.670	0.000*
Stage TNM & Non-classical monocytes TLR-3+ [%]	0.496	4.673	0.000*
Classical monocytes TLR-3+ [%] & sTLR4	0.502	4.745	0.000*
Classical monocytes TLR-3+ [%] & sTLR9	0.502	4.753	0.000*
Classical monocytes TLR-2+ [%] & sTLR4	0.504	4.777	0.000*
Classical monocytes TLR-2+ [%] & sTLR9	0.505	4.792	0.000*
Non-classical monocytes TLR-3+ [%] & sTLR3	0.508	4.826	0.000*
Non-classical monocytes TLR-4+ [%] & sTLR4	0.509	4.846	0.000*
BDCA-1+TLR-2+ [%] & sTLR4	0.510	4.854	0.000*
Non-classical monocytes TLR-4+ [%] & sTLR9	0.511	4.863	0.000*
BDCA-2+TLR-3+ [%] & sTLR4	0.511	4.864	0.000*
BDCA-1+TLR-3+ [%] & sTLR4	0.511	4.868	0.000*
BDCA-1+TLR-4+ [%] & sTLR4	0.512	4.881	0.000*
BDCA-2+TLR-9+ [%] & sTLR4	0.512	4.882	0.000*
Intermediate monocytes in peripheral blood [%] & sTLR4	0.513	4.887	0.000*
BDCA-2+TLR-4+ [%] & sTLR4	0.513	4.891	0.000*
Non-classical monocytes in peripheral blood [%] & sTLR4	0.513	4.891	0.000*
BDCA-1+TLR-2+ [%] & sTLR9	0.513	4.892	0.000*
Classical monocytes TLR-4+ [%] & sTLR4	0.513	4.893	0.000*
BDCA-2+TLR-3+ [%] & sTLR9	0.514	4.900	0.000*
BDCA-1+TLR-3+ [%] & sTLR9	0.514	4.903	0.000*
BDCA-2+TLR-2+ [%] & sTLR4	0.514	4.905	0.000*
Classical monocytes TLR-9+ [%] & sTLR4	0.515	4.914	0.000*
BDCA-1+TLR-4+ [%] & sTLR9	0.515	4.917	0.000*
BDCA-2+TLR-9+ [%] & sTLR9	0.515	4.917	0.000*
Classical monocytes TLR-3+ [%] & sTLR2	0.515	4.920	0.000*
BDCA-1+TLR-9+ [%] & sTLR4	0.515	4.923	0.000*

Intermediate monocytes in peripheral blood [%] & sTLR9	0.516	4.925	0.000*
BDCA-2+TLR-4+ [%] & sTLR9	0.516	4.926	0.000*
Non-classical monocytes in peripheral blood [%] & sTLR9	0.516	4.928	0.000*
Classical monocytes TLR-4+ [%] & sTLR9	0.516	4.928	0.000*
BDCA-2+TLR-2+ [%] & sTLR9	0.517	4.938	0.000*
Classical monocytes TLR-9+ [%] & sTLR9	0.517	4.946	0.000*
BDCA-1+TLR-9+ [%] & sTLR9	0.518	4.962	0.000*
Classical monocytes TLR-2+ [%] & sTLR2	0.522	5.006	0.000*
Non-classical monocytes TLR-4+ [%] & sTLR2	0.522	5.013	0.000*
Stage TNM & Classical monocytes TLR-3+ [%]	0.523	5.023	0.000*
Intermediate monocytes TLR-2 [%] & Intermediate monocytes TLR-4+ [%]	0.525	5.043	0.000*
Stage TNM & Classical monocytes TLR-2+ [%]	0.528	5.095	0.000*
Stage TNM & Non-classical monocytes TLR-4+ [%]	0.528	5.095	0.000*
BDCA-1+TLR-2+ [%] & sTLR2	0.535	5.182	0.000*
BDCA-2+TLR-3+ [%] & sTLR2	0.535	5.183	0.000*
BDCA-1+TLR-3+ [%] & sTLR2	0.535	5.190	0.000*
BDCA-1+TLR-4+ [%] & sTLR2	0.536	5.198	0.000*
Classical monocytes TLR-9+ [%] & sTLR2	0.536	5.201	0.000*
Classical monocytes TLR-3+ [%] & sTLR3	0.537	5.206	0.000*
BDCA-2+TLR-2+ [%] & sTLR2	0.537	5.210	0.000*
BDCA-2+TLR-9+ [%] & sTLR2	0.537	5.212	0.000*
Classical monocytes TLR-4+ [%] & sTLR2	0.537	5.212	0.000*
BDCA-2+TLR-4+ [%] & sTLR2	0.537	5.215	0.000*
Intermediate monocytes in peripheral blood [%] & sTLR2	0.537	5.217	0.000*
Non-classical monocytes in peripheral blood [%] & sTLR2	0.538	5.220	0.000*
Stage TNM & Classical monocytes TLR-9+ [%]	0.539	5.237	0.000*
Stage TNM & BDCA-2+TLR-3+ [%]	0.539	5.243	0.000*
Stage TNM & BDCA-1+TLR-2+ [%]	0.539	5.244	0.000*
BDCA-1+TLR-9+ [%] & sTLR2	0.540	5.247	0.000*
Stage TNM & BDCA-1+TLR-3+ [%]	0.540	5.248	0.000*
Stage TNM & BDCA-1+TLR-4+ [%]	0.540	5.257	0.000*
Stage TNM & Intermediate monocytes in peripheral blood [%]	0.541	5.271	0.000*
Stage TNM & Non-classical monocytes in peripheral blood [%]	0.541	5.271	0.000*

Stage TNM & BDCA-2+TLR-4+ [%]	0.542	5.275	0.000*
Stage TNM & BDCA-2+TLR-9+ [%]	0.542	5.276	0.000*
Stage TNM & BDCA-2+TLR-2+ [%]	0.542	5.279	0.000*
Stage TNM & Classical monocytes TLR-4+ [%]	0.542	5.280	0.000*
sTLR3 & Classical monocytes TLR-2+ [%]	0.542	5.281	0.000*
Non-classical monocytes TLR-4+ [%] & sTLR3	0.544	5.301	0.000*
Stage TNM & BDCA-1+TLR-9+ [%]	0.544	5.311	0.000*
BDCA-1+TLR-2+ [%] & sTLR3	0.554	5.452	0.000*
BDCA-2+TLR-3+ [%] & sTLR3	0.555	5.463	0.000*
BDCA-1+TLR-4+ [%] & sTLR3	0.556	5.472	0.000*
BDCA-1+TLR-3+ [%] & sTLR3	0.557	5.487	0.000*
Classical monocytes TLR-4+ [%] & sTLR3	0.557	5.488	0.000*
Intermediate monocytes in peripheral blood [%] & sTLR3	0.557	5.493	0.000*
BDCA-2+TLR-9+ [%] & sTLR3	0.557	5.493	0.000*
BDCA-2+TLR-4+ [%] & sTLR3	0.557	5.496	0.000*
BDCA-2+TLR-2+ [%] & sTLR3	0.557	5.497	0.000*
Non-classical monocytes in peripheral blood [%] & sTLR3	0.558	5.498	0.000*
BDCA-1+TLR-9+ [%] & sTLR3	0.559	5.523	0.000*
Classical monocytes TLR-9+ [%] & sTLR3	0.560	5.529	0.000*
Intermediate monocytes TLR-3+ [%] & Intermediate monocytes TLR-9+ [%]	0.568	5.644	0.000*
BDCA-2+ occurrence in peripheral blood [%] & Classical monocytes in peripheral blood [%]	0.666	7.301	0.000*
sTLR4 & sTLR3	0.812	11.398	0.000*
sTLR3 & sTLR9	0.818	11.654	0.000*
sTLR2 & sTLR4	0.844	12.905	0.000*
sTLR2 & sTLR9	0.864	14.028	0.000*
Stage TNM & sTLR3	0.869	14.393	0.000*
Stage TNM & sTLR4	0.913	18.301	0.000*
Stage TNM & sTLR9	0.926	20.128	0.000*
BDCA-1+TLR-9+ [%] & Non-classical monocytes TLR-2+ [%]	0.927	20.299	0.000*
BDCA-2+TLR-9+ [%] & Non-classical monocytes TLR-2+ [%]	0.927	20.302	0.000*
Intermediate monocytes in peripheral blood [%] & Non-classical monocytes TLR-2+ [%]	0.927	20.304	0.000*
BDCA-1+TLR-3+ [%] & Non-classical monocytes TLR-2+ [%]	0.927	20.308	0.000*
BDCA-1+TLR-2+ [%] & Non-classical monocytes TLR-2+ [%]	0.928	20.309	0.000*

BDCA-1+TLR-4+ [%] & Non-classical monocytes TLR-2+ [%]	0.928	20.309	0.000*
BDCA-2+TLR-3+ [%] & Non-classical monocytes TLR-2+ [%]	0.928	20.314	0.000*
BDCA-2+TLR-2+ [%] & Non-classical monocytes TLR-2+ [%]	0.928	20.317	0.000*
BDCA-2+TLR-4+ [%] & Non-classical monocytes TLR-2+ [%]	0.928	20.317	0.000*
BDCA-2+TLR-9+ [%] & Non-classical monocytes TLR-9+ [%]	0.929	20.620	0.000*
BDCA-1+TLR-9+ [%] & Non-classical monocytes TLR-9+ [%]	0.929	20.622	0.000*
Intermediate monocytes in peripheral blood [%] & Non-classical monocytes TLR-9+ [%]	0.930	20.630	0.000*
BDCA-1+TLR-3+ [%] & Non-classical monocytes TLR-9+ [%]	0.930	20.631	0.000*
BDCA-1+TLR-2+ [%] & Non-classical monocytes TLR-9+ [%]	0.930	20.631	0.000*
Non-classical monocytes TLR-9+ [%] & BDCA-1+TLR-2+ [%]	0.930	20.631	0.000*
BDCA-2+TLR-3+ [%] & Non-classical monocytes TLR-9+ [%]	0.930	20.637	0.000*
BDCA-2+TLR-2+ [%] & Non-classical monocytes TLR-9+ [%]	0.930	20.637	0.000*
BDCA-2+TLR-4+ [%] & Non-classical monocytes TLR-9+ [%]	0.930	20.640	0.000*
Classical monocytes TLR-4+ [%] & Non-classical monocytes TLR-2+ [%]	0.930	20.676	0.000*
Stage TNM & sTLR2	0.932	20.984	0.000*
Classical monocytes TLR-4+ [%] & Non-classical monocytes TLR-9+ [%]	0.932	21.008	0.000*
Classical monocytes TLR-9+ [%] & Non-classical monocytes TLR-2+ [%]	0.933	21.227	0.000*
Classical monocytes TLR-9+ [%] & Non-classical monocytes TLR-9+ [%]	0.935	21.624	0.000*
sTLR2 & sTLR3	0.939	22.390	0.000*
BDCA-1+TLR-9+ [%] & Non-classical monocytes TLR-3+ [%]	0.946	23.780	0.000*
BDCA-2+TLR-9+ [%] & Non-classical monocytes TLR-3+ [%]	0.946	23.784	0.000*
Intermediate monocytes in peripheral blood [%] & Non-classical monocytes TLR-3+ [%]	0.946	23.786	0.000*
BDCA-1+TLR-3+ [%] & Non-classical monocytes TLR-3+ [%]	0.946	23.791	0.000*
BDCA-1+TLR-2+ [%] & Non-classical monocytes TLR-3+ [%]	0.946	23.792	0.000*
BDCA-2+TLR-3+ [%] & Non-classical monocytes TLR-3+ [%]	0.946	23.792	0.000*
BDCA-1+TLR-4+ [%] & Non-classical monocytes TLR-3+ [%]	0.946	23.794	0.000*
BDCA-2+TLR-2+ [%] & Non-classical monocytes TLR-3+ [%]	0.946	23.801	0.000*
BDCA-2+TLR-4+ [%] & Non-classical monocytes TLR-3+ [%]	0.946	23.805	0.000*
Classical monocytes TLR-4+ [%] & Non-classical monocytes TLR-3+ [%]	0.948	24.281	0.000*
Classical monocytes TLR-9+ [%] & Non-classical monocytes TLR-3+ [%]	0.949	24.580	0.000*
Non-classical monocytes TLR-2+ [%] & Non-classical monocytes TLR-4+ [%]	0.962	28.642	0.000*
Non-classical monocytes TLR-9+ [%] & Non-classical monocytes TLR-4+ [%]	0.963	29.159	0.000*
Classical monocytes TLR-2+ [%] & Non-classical monocytes TLR-2+ [%]	0.971	33.037	0.000*

Classical monocytes TLR-2+ [%] & Non-classical monocytes TLR-9+ [%]	0.972	33.797	0.000*
Non-classical monocytes TLR-3+ [%] & Non-classical monocytes TLR-4+ [%]	0.974	34.851	0.000*
Classical monocytes TLR-3+ [%] & Non-classical monocytes TLR-2+ [%]	0.980	39.828	0.000*
Classical monocytes TLR-3+ [%] & Non-classical monocytes TLR-9+ [%]	0.980	40.796	0.000*
Classical monocytes TLR-2+ [%] & Non-classical monocytes TLR-3+ [%]	0.982	42.375	0.000*
BDCA-2+TLR-9+ [%] & Classical monocytes TLR-3+ [%]	0.983	43.545	0.000*
BDCA-2+TLR-3+ [%] & Classical monocytes TLR-3+ [%]	0.983	43.570	0.000*
BDCA-1+TLR-9+ [%] & Classical monocytes TLR-3+ [%]	0.983	43.581	0.000*
BDCA-1+TLR-2+ [%] & Classical monocytes TLR-3+ [%]	0.983	43.582	0.000*
BDCA-1+TLR-4+ [%] & Classical monocytes TLR-3+ [%]	0.983	43.606	0.000*
Non-classical monocytes in peripheral blood [%] & Classical monocytes TLR-3+ [%]	0.983	43.631	0.000*
BDCA-2+TLR-2+ [%] & Classical monocytes TLR-3+ [%]	0.983	43.654	0.000*
BDCA-1+TLR-3+ [%] & Classical monocytes TLR-3+ [%]	0.983	43.665	0.000*
BDCA-2+TLR-4+ [%] & Classical monocytes TLR-3+ [%]	0.983	43.666	0.000*
Classical monocytes TLR-3+ [%] & Classical monocytes TLR-4+ [%]	0.984	45.094	0.000*
Classical monocytes TLR-3+ [%] & Classical monocytes TLR-9+ [%]	0.984	45.200	0.000*
Classical monocytes TLR-3+ [%] & Non-classical monocytes TLR-3+ [%]	0.989	54.389	0.000*
BDCA-2+TLR-9+ [%] & Classical monocytes TLR-2+ [%]	0.990	56.755	0.000*
BDCA-1+TLR-9+ [%] & Classical monocytes TLR-2+ [%]	0.990	56.807	0.000*
BDCA-1+TLR-3+ [%] & Classical monocytes TLR-2+ [%]	0.990	56.833	0.000*
Intermediate monocytes in peripheral blood [%] & Classical monocytes TLR-2+ [%]	0.990	56.861	0.000*
Non-classical monocytes in peripheral blood [%] & Classical monocytes TLR-2+ [%]	0.990	56.861	0.000*
BDCA-1+TLR-2+ [%] & Classical monocytes TLR-2+ [%]	0.990	56.912	0.000*
BDCA-1+TLR-4+ [%] & Classical monocytes TLR-2+ [%]	0.990	56.912	0.000*
BDCA-2+TLR-3+ [%] & Classical monocytes TLR-2+ [%]	0.990	56.938	0.000*
BDCA-2+TLR-2+ [%] & Classical monocytes TLR-2+ [%]	0.990	56.990	0.000*
BDCA-2+TLR-4+ [%] & Classical monocytes TLR-2+ [%]	0.990	57.043	0.000*
Classical monocytes TLR-2+ [%] & Classical monocytes TLR-4+ [%]	0.991	59.454	0.000*
Classical monocytes TLR-9+ [%] & Classical monocytes TLR-2+ [%]	0.991	59.633	0.000*
BDCA-1+TLR-9+ [%] & Non-classical monocytes TLR-4+ [%]	0.994	71.825	0.000*
BDCA-2+TLR-9+ [%] & Non-classical monocytes TLR-4+ [%]	0.994	71.928	0.000*
BDCA-1+TLR-3+ [%] & Non-classical monocytes TLR-4+ [%]	0.994	72.084	0.000*
Intermediate monocytes in peripheral blood [%] & Non-classical monocytes TLR-4+ [%]	0.994	72.140	0.000*

BDCA-1+TLR-2+ [%] & Non-classical monocytes TLR-4+ [%]	0.994	72.242	0.000*
Non-classical monocytes TLR-4+ [%] & BDCA-1+TLR-2+ [%]	0.994	72.242	0.000*
BDCA-2+TLR-3+ [%] & Non-classical monocytes TLR-4+ [%]	0.994	72.400	0.000*
BDCA-2+TLR-2+ [%] & Non-classical monocytes TLR-4+ [%]	0.994	72.401	0.000*
BDCA-2+TLR-4+ [%] & Non-classical monocytes TLR-4+ [%]	0.994	72.507	0.000*
Classical monocytes TLR-4+ [%] & Non-classical monocytes TLR-4+ [%]	0.994	76.212	0.000*
Classical monocytes TLR-9+ [%] & Non-classical monocytes TLR-4+ [%]	0.995	81.055	0.000*
Classical monocytes TLR-3+ [%] & Non-classical monocytes TLR-4+ [%]	0.996	89.794	0.000*
sTLR4 & sTLR9	0.997	101.828	0.000*
Non-classical monocytes TLR-3+ [%] & Non-classical monocytes TLR-2+ [%]	0.998	123.930	0.000*
Non-classical monocytes TLR-3+ [%] & Non-classical monocytes TLR-9+ [%]	0.998	126.628	0.000*
Classical monocytes TLR-2+ [%] & Non-classical monocytes TLR-4+ [%]	0.998	147.144	0.000*
BDCA-2+TLR-9+ [%] & Classical monocytes TLR-9+ [%]	0.999	160.545	0.000*
BDCA-1+TLR-9+ [%] & Classical monocytes TLR-9+ [%]	0.999	160.546	0.000*
BDCA-1+TLR-3+ [%] & Classical monocytes TLR-9+ [%]	0.999	162.273	0.000*
Intermediate monocytes in peripheral blood [%] & Classical monocytes TLR-9+ [%]	0.999	162.868	0.000*
BDCA-1+TLR-2+ [%] & Classical monocytes TLR-9+ [%]	0.999	164.059	0.000*
Classical monocytes TLR-9+ [%] & BDCA-1+TLR-2+ [%]	0.999	164.059	0.000*
BDCA-2+TLR-3+ [%] & Classical monocytes TLR-9+ [%]	0.999	164.667	0.000*
BDCA-2+TLR-2+ [%] & Classical monocytes TLR-9+ [%]	0.999	165.904	0.000*
BDCA-2+TLR-4+ [%] & Classical monocytes TLR-9+ [%]	0.999	167.170	0.000*
Classical monocytes TLR-3+ [%] & Classical monocytes TLR-2+ [%]	0.999	169.781	0.000*
Classical monocytes TLR-9+ [%] & Classical monocytes TLR-4+ [%]	0.999	170.458	0.000*
BDCA-1+TLR-9+ [%] & BDCA-2+TLR-9+ [%]	1.000	355.514	0.000*
Non-classical monocytes TLR-9+ [%] & Non-classical monocytes TLR-2+ [%]	1.000	390.836	0.000*
BDCA-1+TLR-3+ [%] & BDCA-2+TLR-9+ [%]	1.000	399.220	0.000*
BDCA-1+TLR-9+ [%] & BDCA-2+TLR-3+ [%]	1.000	399.231	0.000*
BDCA-2+TLR-9+ [%] & Classical monocytes TLR-4+ [%]	1.000	408.207	0.000*
BDCA-1+TLR-9+ [%] & Classical monocytes TLR-4+ [%]	1.000	408.209	0.000*
Intermediate monocytes in peripheral blood [%] & BDCA-2+TLR-9+ [%]	1.000	408.218	0.000*
Intermediate monocytes in peripheral blood [%] & BDCA-1+TLR-9+ [%]	1.000	408.219	0.000*
Classical monocytes TLR-4+ [%] & BDCA-1+TLR-3+ [%]	1.000	417.820	0.000*
BDCA-2+TLR-9+ [%] & BDCA-1+TLR-2+ [%]	1.000	428.132	0.000*

BDCA-2+TLR-9+ [%] & BDCA-1+TLR-4+ [%]	1.000	428.132	0.000*
Non-classical monocytes in peripheral blood [%] & BDCA-1+TLR-9+ [%]	1.000	428.150	0.000*
Intermediate monocytes in peripheral blood [%] & Classical monocytes TLR-4+ [%]	1.000	428.158	0.000*
Non-classical monocytes in peripheral blood [%] & Classical monocytes TLR-4+ [%]	1.000	428.158	0.000*
BDCA-1+TLR-3+ [%] & BDCA-1+TLR-9+ [%]	1.000	439.253	0.000*
BDCA-2+TLR-3+ [%] & BDCA-2+TLR-9+ [%]	1.000	439.260	0.000*
BDCA-1+TLR-4+ [%] & BDCA-1+TLR-9+ [%]	1.000	439.260	0.000*
BDCA-1+TLR-3+ [%] & BDCA-1+TLR-2+ [%]	1.000	439.262	0.000*
BDCA-1+TLR-3+ [%] & BDCA-1+TLR-4+ [%]	1.000	451.301	0.000*
BDCA-1+TLR-4+ [%] & Classical monocytes TLR-4+ [%]	1.000	451.311	0.000*
Intermediate monocytes in peripheral blood [%] & BDCA-1+TLR-3+ [%]	1.000	464.406	0.000*
Non-classical monocytes in peripheral blood [%] & BDCA-1+TLR-3+ [%]	1.000	464.406	0.000*
Non-classical monocytes in peripheral blood [%] & BDCA-2+TLR-3+ [%]	1.000	464.411	0.000*
BDCA-1+TLR-9+ [%] & BDCA-1+TLR-2+ [%]	1.000	478.682	0.000*
BDCA-1+TLR-2+ [%] & BDCA-1+TLR-4+ [%]	1.000	478.690	0.000*
BDCA-1+TLR-9+ [%] & BDCA-2+TLR-2+ [%]	1.000	478.691	0.000*
BDCA-1+TLR-2+ [%] & Classical monocytes TLR-4+ [%]	1.000	478.695	0.000*
Intermediate monocytes in peripheral blood [%] & BDCA-1+TLR-2+ [%]	1.000	478.706	0.000*
Non-classical monocytes in peripheral blood [%] & BDCA-1+TLR-2+ [%]	1.000	478.706	0.000*
BDCA-2+TLR-9+ [%] & BDCA-2+TLR-2+ [%]	1.000	494.392	0.000*
BDCA-2+TLR-4+ [%] & BDCA-2+TLR-9+ [%]	1.000	494.399	0.000*
Intermediate monocytes in peripheral blood [%] & BDCA-2+TLR-3+ [%]	1.000	494.410	0.000*
BDCA-1+TLR-3+ [%] & BDCA-2+TLR-3+ [%]	1.000	511.745	0.000*
BDCA-1+TLR-3+ [%] & BDCA-2+TLR-2+ [%]	1.000	511.751	0.000*
BDCA-2+TLR-2+ [%] & Classical monocytes TLR-4+ [%]	1.000	511.760	0.000*
Intermediate monocytes in peripheral blood [%] & BDCA-2+TLR-2+ [%]	1.000	511.770	0.000*
BDCA-1+TLR-2+ [%] & BDCA-2+TLR-3+ [%]	1.000	531.072	0.000*
BDCA-2+TLR-3+ [%] & Classical monocytes TLR-4+ [%]	1.000	531.077	0.000*
BDCA-1+TLR-2+ [%] & BDCA-2+TLR-2+ [%]	1.000	531.078	0.000*
Non-classical monocytes in peripheral blood [%] & BDCA-2+TLR-2+ [%]	1.000	531.093	0.000*
BDCA-1+TLR-4+ [%] & BDCA-2+TLR-2+ [%]	1.000	552.767	0.000*
BDCA-1+TLR-3+ [%] & BDCA-2+TLR-4+ [%]	1.000	552.771	0.000*
Intermediate monocytes in peripheral blood [%] & BDCA-1+TLR-4+ [%]	1.000	552.778	0.000*

Non-classical monocytes in peripheral blood [%] & BDCA-1+TLR-4+ [%]	1.000	552.778	0.000*
BDCA-1+TLR-4+ [%] & BDCA-2+TLR-3+ [%]	1.000	577.344	0.000*
BDCA-2+TLR-3+ [%] & BDCA-2+TLR-2+ [%]	1.000	577.353	0.000*
BDCA-1+TLR-9+ [%] & BDCA-2+TLR-4+ [%]	1.000	577.354	0.000*
Intermediate monocytes in peripheral blood [%] & BDCA-2+TLR-4+ [%]	1.000	577.371	0.000*
BDCA-1+TLR-4+ [%] & BDCA-2+TLR-4+ [%]	1.000	638.303	0.000*
BDCA-2+TLR-4+ [%] & Classical monocytes TLR-4+ [%]	1.000	638.306	0.000*
Classical monocytes TLR-4+ [%] & BDCA-2+TLR-4+ [%]	1.000	638.306	0.000*
Non-classical monocytes in peripheral blood [%] & BDCA-2+TLR-4+ [%]	1.000	638.316	0.000*
BDCA-2+TLR-3+ [%] & BDCA-2+TLR-4+ [%]	1.000	677.029	0.000*
BDCA-1+TLR-2+ [%] & BDCA-2+TLR-4+ [%]	1.000	723.778	0.000*
BDCA-2+TLR-4+ [%] & BDCA-2+TLR-2+ [%]	1.000	781.781	0.000*
Intermediate monocytes in peripheral blood [%] & Non-classical monocytes in peripheral blood [%]	1.000	1354.144	0.000*

The symbol * indicates statistically significant results.

Supplementary Materials Table S6- ROC curve analysis for disease stage of GC patients

	BDCA-1+TLR-3+ [%]					
	1 vs. 2	1 vs. 3	1 vs. 4	2 vs. 3	2 vs. 4	3 vs. 4
Area	0.6958	0.8569	0.8746	0.7279	0.753	0.5854
Std. Error	0.0962	0.06989	0.0582	0.08887	0.0804	0.09365
95% confidence interval	0.5073 to 0.8844	0.7199 to 0.9938	0.7605 to 0.9887	0.5538 to 0.9021	0.5954 to 0.9106	0.4019 to 0.7690
p-Value	0.0632	0.0006*	0.0002*	0.0255*	0.0092*	0.3706
	BDCA-1+TLR-9+[%]					
	1 vs. 2	1 vs. 3	1 vs. 4	2 vs. 3	2 vs. 4	3 vs. 4
Area	0.7021	0.8569	0.8778	0.7261	0.7574	0.5854
Std. Error	0.09541	0.06952	0.05747	0.08912	0.07971	0.09355
95% confidence interval	0.5151 to 0.8891	0.7206 to 0.9931	0.7651 to 0.9904	0.5514 to 0.9008	0.6012 to 0.9137	0.4021 to 0.7688
p-Value	0.0552	0.0006*	0.0001*	0.0267*	0.008*	0.3706

	BDCA-1+TLR-2+[%]					
	1 vs. 2	1 vs. 3	1 vs. 4	2 vs. 3	2 vs. 4	3 vs. 4
Area	0.7	0.8549	0.8746	0.7243	0.753	0.5868
Std. Error	0.09569	0.07072	0.0586	0.08925	0.08025	0.09363
95% confidence interval	0.5125 to 0.8875	0.7163 to 0.9935	0.7598 to 0.9895	0.5493 to 0.8992	0.5957 to 0.9103	0.4033 to 0.7703
p-Value	0.0578	0.0006*	0.0002*	0.028*	0.0092*	0.3628
	BDCA-1+TLR-4+[%]					
	1 vs. 2	1 vs. 3	1 vs. 4	2 vs. 3	2 vs. 4	3 vs. 4
Area	0.7	0.8549	0.8762	0.7206	0.756	0.584
Std. Error	0.09569	0.07072	0.05791	0.0899	0.07996	0.09369
95% confidence interval	0.5125 to 0.8875	0.7163 to 0.9935	0.7627 to 0.9897	0.5444 to 0.8968	0.5992 to 0.9127	0.4004 to 0.7677
p-Value	0.0578	0.0006*	0.0001*	0.0307*	0.0084*	0.3785
	BDCA-2+TLR-3+[%]					
	1 vs. 2	1 vs. 3	1 vs. 4	2 vs. 3	2 vs. 4	3 vs. 4
Area	0.7	0.8549	0.8762	0.7224	0.753	0.584
Std. Error	0.09569	0.07072	0.05791	0.08957	0.08046	0.09377
95% confidence interval	0.5125 to 0.8875	0.7163 to 0.9935	0.7627 to 0.9897	0.5469 to 0.8980	0.5953 to 0.9107	0.4002 to 0.7678
p-Value	0.0578	0.0006*	0.0001*	0.0293*	0.0092*	0.3785
	BDCA-2+TLR-9+[%]					
	1 vs. 2	1 vs. 3	1 vs. 4	2 vs. 3	2 vs. 4	3 vs. 4
Area	0.7	0.8549	0.8762	0.7243	0.7574	0.584
Std. Error	0.09569	0.07072	0.05791	0.08937	0.07971	0.09377
95% confidence interval	0.5125 to 0.8875	0.7163 to 0.9935	0.7627 to 0.9897	0.5491 to 0.8994	0.6012 to 0.9137	0.4002 to 0.7678
p-Value	0.0578	0.0006*	0.0001*	0.028*	0.008*	0.3785
	BDCA-2+TLR-2+[%]					
	1 vs. 2	1 vs. 3	1 vs. 4	2 vs. 3	2 vs. 4	3 vs. 4

Area	0.7	0.8549	0.8762	0.7279	0.756	0.5854
Std. Error	0.09569	0.07072	0.05791	0.08908	0.07995	0.09365
95% confidence interval	0.5125 to 0.8875	0.7163 to 0.9935	0.7627 to 0.9897	0.5533 to 0.9025	0.5992 to 0.9127	0.4019 to 0.7690
p-Value	0.0578	0.0006*	0.0001*	0.0255*	0.0084*	0.3706
	BDCA-2+TLR-4+[%]					
	1 vs. 2	1 vs. 3	1 vs. 4	2 vs. 3	2 vs. 4	3 vs. 4
Area	0.7	0.8549	0.8762	0.7261	0.756	0.5854
Std. Error	0.09569	0.07072	0.05791	0.08912	0.07995	0.09365
95% confidence interval	0.5125 to 0.8875	0.7163 to 0.9935	0.7627 to 0.9897	0.5514 to 0.9008	0.5992 to 0.9127	0.4019 to 0.7690
p-Value	0.0578	0.0006*	0.0001*	0.0267*	0.0084*	0.3706
	TLR-3+ on classical monocytes [%]					
	1 vs. 2	1 vs. 3	1 vs. 4	2 vs. 3	2 vs. 4	3 vs. 4
Area	0.6604	0.8412	0.8587	0.7279	0.7589	0.5854
Std. Error	0.1005	0.07497	0.06211	0.08887	0.07958	0.09365
95% confidence interval	0.4635 to 0.8573	0.6942 to 0.9881	0.7370 to 0.9805	0.5538 to 0.9021	0.6030 to 0.9149	0.4019 to 0.7690
p-Value	0.128	0.001*	0.0003*	0.0255*	0.0076*	0.3706
	TLR-9+ on classical monocytes [%]					
	1 vs. 2	1 vs. 3	1 vs. 4	2 vs. 3	2 vs. 4	3 vs. 4
Area	0.6979	0.8549	0.8762	0.7243	0.756	0.577
Std. Error	0.09624	0.07072	0.05791	0.08937	0.07995	0.09443
95% confidence interval	0.5093 to 0.8865	0.7163 to 0.9935	0.7627 to 0.9897	0.5491 to 0.8994	0.5992 to 0.9127	0.3920 to 0.7621
p-Value	0.0604	0.0006*	0.0001*	0.028*	0.0084*	0.4195
	TLR-2+ on classical monocytes [%]					
	1 vs. 2	1 vs. 3	1 vs. 4	2 vs. 3	2 vs. 4	3 vs. 4
Area	0.675	0.8431	0.8667	0.7243	0.756	0.5854
Std. Error	0.09967	0.07536	0.06111	0.08937	0.07995	0.09365

95% confidence interval	0.4796 to 0.8704	0.6954 to 0.9908	0.7469 to 0.9864	0.5491 to 0.8994	0.5992 to 0.9127	0.4019 to 0.7690
p-Value	0.0969	0.001*	0.0002*	0.028*	0.0084*	0.3706
	TLR-4+ on classical monocytes [%]					
	1 vs. 2	1 vs. 3	1 vs. 4	2 vs. 3	2 vs. 4	3 vs. 4
Area	0.7	0.8549	0.8762	0.7243	0.7574	0.5854
Std. Error	0.09569	0.07072	0.05786	0.08937	0.07955	0.09365
95% confidence interval	0.5125 to 0.8875	0.7163 to 0.9935	0.7628 to 0.9896	0.5491 to 0.8994	0.6015 to 0.9134	0.4019 to 0.7690
p-Value	0.0578	0.0006*	0.0001*	0.028*	0.008*	0.3706
	TLR-3+ on intermediate monocytes [%]					
	1 vs. 2	1 vs. 3	1 vs. 4	2 vs. 3	2 vs. 4	3 vs. 4
Area	0.5708	0.6039	0.5778	0.5037	0.6429	0.6583
Std. Error	0.1064	0.1011	0.09852	0.1047	0.09228	0.09018
95% confidence interval	0.3623 to 0.7793	0.4058 to 0.8021	0.3847 to 0.7709	0.2985 to 0.7088	0.4620 to 0.8237	0.4815 to 0.8350
p-Value	0.5016	0.317	0.4318	0.9713	0.1411	0.0972
	TLR-9+ on intermediate monocytes [%]					
	1 vs. 2	1 vs. 3	1 vs. 4	2 vs. 3	2 vs. 4	3 vs. 4
Area	0.575	0.6216	0.6984	0.5037	0.622	0.6106
Std. Error	0.105	0.1012	0.09067	0.1041	0.0945	0.09537
95% confidence interval	0.3692 to 0.7808	0.4232 to 0.8199	0.5207 to 0.8761	0.2996 to 0.7077	0.4368 to 0.8072	0.4237 to 0.7976
p-Value	0.4768	0.2417	0.0449	0.9713	0.2088	0.2462
	TLR-2+ on intermediate monocytes [%]					
	1 vs. 2	1 vs. 3	1 vs. 4	2 vs. 3	2 vs. 4	3 vs. 4
Area	0.5292	0.5137	0.5397	0.5257	0.6161	0.5966
Std. Error	0.1115	0.1073	0.1077	0.1036	0.09546	0.09928
95% confidence interval	0.3106 to 0.7477	0.3034 to 0.7241	0.3286 to 0.7507	0.3228 to 0.7287	0.4290 to 0.8032	0.4021 to 0.7912

p-Value	0.782	0.8949	0.6884	0.8009	0.2318	0.3111
	TLR-4+ on intermediate monocytes [%]					
	1 vs. 2	1 vs. 3	1 vs. 4	2 vs. 3	2 vs. 4	3 vs. 4
Area	0.5313	0.6392	0.581	0.6324	0.5595	0.5882
Std. Error	0.1075	0.1034	0.1081	0.09876	0.1008	0.09492
95% confidence interval	0.3206 to 0.7419	0.4366 to 0.8419	0.3690 to 0.7929	0.4388 to 0.8259	0.3619 to 0.7571	0.4022 to 0.7743
p-Value	0.7669	0.1801	0.4132	0.1947	0.5398	0.3551
	TLR-3+ on non-classical monocytes [%]					
	1 vs. 2	1 vs. 3	1 vs. 4	2 vs. 3	2 vs. 4	3 vs. 4
Area	0.6354	0.8	0.8429	0.7243	0.7574	0.5854
Std. Error	0.1024	0.08374	0.06551	0.08937	0.07971	0.09365
95% confidence interval	0.4347 to 0.8361	0.6359 to 0.9641	0.7145 to 0.9713	0.5491 to 0.8994	0.6012 to 0.9137	0.4019 to 0.7690
p-Value	0.1989	0.0039*	0.0005*	0.028*	0.008*	0.3706
	TLR-9+ on non-classical monocytes [%]					
	1 vs. 2	1 vs. 3	1 vs. 4	2 vs. 3	2 vs. 4	3 vs. 4
Area	0.6313	0.798	0.8222	0.7224	0.756	0.577
Std. Error	0.1034	0.08413	0.07338	0.08957	0.07995	0.09443
95% confidence interval	0.4286 to 0.8339	0.6331 to 0.9629	0.6784 to 0.9660	0.5469 to 0.8980	0.5992 to 0.9127	0.3920 to 0.7621
p-Value	0.2131	0.0041*	0.0011*	0.0293*	0.0084*	0.4195
	TLR-2+ on non-classical monocytes [%]					
	1 vs. 2	1 vs. 3	1 vs. 4	2 vs. 3	2 vs. 4	3 vs. 4
Area	0.6313	0.798	0.819	0.7261	0.756	0.5756
Std. Error	0.1032	0.08413	0.07492	0.08919	0.07995	0.09453
95% confidence interval	0.4290 to 0.8335	0.6331 to 0.9629	0.6722 to 0.9659	0.5513 to 0.9009	0.5992 to 0.9127	0.3904 to 0.7609
p-Value	0.2131	0.0041*	0.0013*	0.0267*	0.0084*	0.428

	TLR-4+ on non-classical monocytes [%]					
	1 vs. 2	1 vs. 3	1 vs. 4	2 vs. 3	2 vs. 4	3 vs. 4
Area	0.6792	0.8431	0.8698	0.7243	0.756	0.5756
Std. Error	0.09948	0.07536	0.06054	0.08937	0.07995	0.09445
95% confidence interval	0.4842 to 0.8741	0.6954 to 0.9908	0.7512 to 0.9885	0.5491 to 0.8994	0.5992 to 0.9127	0.3905 to 0.7608
p-Value	0.0892	0.001*	0.0002*	0.028*	0.0084*	0.428
	Serum concentration of sTLR-2					
	1 vs. 2	1 vs. 3	1 vs. 4	2 vs. 3	2 vs. 4	3 vs. 4
Area	1	1	1	0.8235	1	0.9804
Std. Error	0	0	0	0.07724	0	0.01682
95% confidence interval	1.000 to 1.000	1.000 to 1.000	1.000 to 1.000	0.6721 to 0.9749	1.000 to 1.000	0.9474 to 1.000
p-Value	<0.0001*	<0.0001*	<0.0001*	0.0015*	<0.0001*	<0.0001*
	Serum concentration of sTLR-4					
	1 vs. 2	1 vs. 3	1 vs. 4	2 vs. 3	2 vs. 4	3 vs. 4
Area	0.9542	1	1	0.9963	1	0.8179
Std. Error	0.03548	0	0	0.006109	0	0.06848
95% confidence interval	0.8846 to 1.000	1.000 to 1.000	1.000 to 1.000	0.9844 to 1.000	1.000 to 1.000	0.6837 to 0.9521
p-Value	<0.0001*	<0.0001*	<0.0001*	<0.0001*	<0.0001*	0.0009*
	Serum concentration of sTLR-3					
	1 vs. 2	1 vs. 3	1 vs. 4	2 vs. 3	2 vs. 4	3 vs. 4
Area	0.9667	1	1	1	1	0.6387
Std. Error	0.02673	0	0	0	0	0.08988
95% confidence interval	0.9143 to 1.000	1.000 to 1.000	1.000 to 1.000	1.000 to 1.000	1.000 to 1.000	0.4625 to 0.8148
p-Value	<0.0001*	<0.0001*	<0.0001*	<0.0001*	<0.0001*	0.1462
	Serum concentration of sTLR-9					
	1 vs. 2	1 vs. 3	1 vs. 4	2 vs. 3	2 vs. 4	3 vs. 4

Area	1	1	1	0.9743	1	0.8599
Std. Error	0	0	0	0.02176	0	0.05869
95% confidence interval	1.000 to 1.000	1.000 to 1.000	1.000 to 1.000	0.9316 to 1.000	1.000 to 1.000	0.7449 to 0.9750
p-Value	<0.0001*	<0.0001*	<0.0001*	<0.0001*	<0.0001*	0.0002*

Supplementary Materials Table S7- ROC curve analysis for gender of GC and HV patients

	BDCA-1+TLR-3+ [%]					
	1 vs. 2	1 vs. 3	1 vs. 4	2 vs. 3	2 vs. 4	3 vs. 4
Area	0.5361	0.7393	0.744	0.7984	0.8049	0.5444
Std. Error	0.07217	0.07941	0.07495	0.07086	0.06377	0.1099
95% confidence interval	0.3947 to 0.6776	0.5836 to 0.8949	0.5972 to 0.8909	0.6595 to 0.9372	0.6799 to 0.9299	0.3290 to 0.7599
p-Value	0.6121	0.0104*	0.009*	0.0007*	0.0005*	0.6783
	BDCA-1+TLR-9+ [%]					
	1 vs. 2	1 vs. 3	1 vs. 4	2 vs. 3	2 vs. 4	3 vs. 4
Area	0.5375	0.894	0.8786	0.9366	0.922	0.5378
Std. Error	0.07208	0.04675	0.05162	0.03332	0.03832	0.1078
95% confidence interval	0.3962 to 0.6787	0.8024 to 0.9857	0.7774 to 0.9797	0.8713 to 1.000	0.8468 to 0.9971	0.3266 to 0.7490
p-Value	0.5993	<0.0001*	<0.0001*	<0.0001*	<0.0001*	0.7244
	BDCA-1+TLR-2+ [%]					
	1 vs. 2	1 vs. 3	1 vs. 4	2 vs. 3	2 vs. 4	3 vs. 4
Area	0.5344	1	1	1	1	0.5711
Std. Error	0.0721	0	0	0	0	0.107
95% confidence interval	0.3931 to 0.6757	1.000 to 1.000	1.000 to 1.000	1.000 to 1.000	1.000 to 1.000	0.3613 to 0.7809
p-Value	0.6293	<0.0001*	<0.0001*	<0.0001*	<0.0001*	0.5069
	BDCA-1+TLR-4+ [%]					
	1 vs. 2	1 vs. 3	1 vs. 4	2 vs. 3	2 vs. 4	3 vs. 4

Area	0.534	0.9929	0.9952	1	0.9984	0.5422
Std. Error	0.07213	0.008105	0.006443	0	0.00273	0.1075
95% confidence interval	0.3926 to 0.6753	0.9770 to 1.000	0.9826 to 1.000	1.000 to 1.000	0.9930 to 1.000	0.3314 to 0.7530
p-Value	0.6337	<0.0001*	<0.0001*	<0.0001*	<0.0001*	0.6936
	BDCA-2+TLR-3+[%]					
	1 vs. 2	1 vs. 3	1 vs. 4	2 vs. 3	2 vs. 4	3 vs. 4
Area	0.5335	0.775	0.7381	0.8569	0.8	0.52
Std. Error	0.07215	0.07285	0.07581	0.05221	0.06418	0.1127
95% confidence interval	0.3921 to 0.6749	0.6322 to 0.9178	0.5895 to 0.8867	0.7546 to 0.9592	0.6742 to 0.9258	0.2991 to 0.7409
p-Value	0.638	0.0032*	0.0108*	<0.0001*	0.0006*	0.8519
	BDCA-2+TLR-9+[%]					
	1 vs. 2	1 vs. 3	1 vs. 4	2 vs. 3	2 vs. 4	3 vs. 4
Area	0.5335	0.95	0.9405	0.9854	0.9707	0.52
Std. Error	0.0722	0.03246	0.03359	0.01183	0.01892	0.1127
95% confidence interval	0.3920 to 0.6751	0.8864 to 1.000	0.8746 to 1.000	0.9622 to 1.000	0.9337 to 1.000	0.2991 to 0.7409
p-Value	0.638	<0.0001*	<0.0001*	<0.0001*	<0.0001*	0.8519
	BDCA-2+TLR-2+[%]					
	1 vs. 2	1 vs. 3	1 vs. 4	2 vs. 3	2 vs. 4	3 vs. 4
Area	0.5348	0.9952	0.9881	0.9984	0.9951	0.52
Std. Error	0.07211	0.006443	0.0115	0.00273	0.005571	0.1127
95% confidence interval	0.3935 to 0.6762	0.9826 to 1.000	0.9656 to 1.000	0.9930 to 1.000	0.9842 to 1.000	0.2991 to 0.7409
p-Value	0.625	<0.0001*	<0.0001*	<0.0001*	<0.0001*	0.8519
	BDCA-2+TLR-4+ [%]					
	1 vs. 2	1 vs. 3	1 vs. 4	2 vs. 3	2 vs. 4	3 vs. 4
Area	0.5348	1	1	1	1	0.52
Std. Error	0.07213	0	0	0	0	0.1127

95% confidence interval	0.3935 to 0.6762	1.000 to 1.000	1.000 to 1.000	1.000 to 1.000	1.000 to 1.000	0.2991 to 0.7409
p-Value	0.625	<0.0001*	<0.0001*	<0.0001*	<0.0001*	0.8519
	TLR-3+ on classical monocytes [%]					
	1 vs. 2	1 vs. 3	1 vs. 4	2 vs. 3	2 vs. 4	3 vs. 4
Area	0.52	0.531	0.5583	0.5528	0.5561	0.6756
Std. Error	0.07167	0.09121	0.09413	0.08354	0.08789	0.1031
95% confidence interval	0.3796 to 0.6605	0.3522 to 0.7097	0.3738 to 0.7428	0.3891 to 0.7166	0.3838 to 0.7284	0.4735 to 0.8776
p-Value	0.7787	0.7404	0.5324	0.5476	0.5233	0.1013
	TLR-9+ on classical monocytes [%]					
	1 vs. 2	1 vs. 3	1 vs. 4	2 vs. 3	2 vs. 4	3 vs. 4
Area	0.5318	0.6286	0.6833	0.6634	0.7285	0.68
Std. Error	0.07221	0.08434	0.08037	0.07071	0.06667	0.1062
95% confidence interval	0.3903 to 0.6733	0.4633 to 0.7939	0.5258 to 0.8409	0.5248 to 0.8020	0.5978 to 0.8591	0.4719 to 0.8881
p-Value	0.6556	0.1688	0.0497*	0.063	0.0093*	0.093*
	TLR-2+ on classical monocytes [%]					
	1 vs. 2	1 vs. 3	1 vs. 4	2 vs. 3	2 vs. 4	3 vs. 4
Area	0.5218	0.9929	0.9881	0.9935	0.9902	0.68
Std. Error	0.07168	0.008105	0.01201	0.006868	0.009144	0.1062
95% confidence interval	0.3813 to 0.6623	0.9770 to 1.000	0.9646 to 1.000	0.9800 to 1.000	0.9723 to 1.000	0.4719 to 0.8881
p-Value	0.76	<0.0001*	<0.0001*	<0.0001*	<0.0001*	0.093
	TLR-4+ on classical monocytes[%]					
	1 vs. 2	1 vs. 3	1 vs. 4	2 vs. 3	2 vs. 4	3 vs. 4
Area	0.5348	0.931	0.95	0.9886	0.9854	0.68
Std. Error	0.0721	0.04035	0.03034	0.009882	0.0119	0.1062
95% confidence interval	0.3935 to 0.6762	0.8519 to 1.000	0.8905 to 1.000	0.9692 to 1.000	0.9620 to 1.000	0.4719 to 0.8881

p-Value	0.625	<0.0001*	<0.0001*	<0.0001*	<0.0001*	0.093
	TLR-3+ on intermediate monocytes[%]					
	1 vs. 2	1 vs. 3	1 vs. 4	2 vs. 3	2 vs. 4	3 vs. 4
Area	0.5083	0.8583	0.9095	0.8537	0.9106	0.6244
Std. Error	0.07277	0.05888	0.04941	0.05163	0.039	0.1056
95% confidence interval	0.3656 to 0.6509	0.7429 to 0.9737	0.8127 to 1.000	0.7525 to 0.9548	0.8341 to 0.9870	0.4174 to 0.8315
p-Value	0.9076	0.0001*	<0.0001*	<0.0001*	<0.0001*	0.2455
	TLR-9+ on intermediate monocytes[%]					
	1 vs. 2	1 vs. 3	1 vs. 4	2 vs. 3	2 vs. 4	3 vs. 4
Area	0.544	0.8667	0.8929	0.8929	0.9252	0.6222
Std. Error	0.0715	0.05626	0.05208	0.05208	0.03521	0.1056
95% confidence interval	0.4039 to 0.6841	0.7564 to 0.9769	0.7908 to 0.9949	0.7908 to 0.9949	0.8562 to 0.9942	0.4152 to 0.8293
p-Value	0.5372	<0.0001*	<0.0001*	<0.0001*	<0.0001*	0.254
	TLR-2+ on intermediate monocytes[%]					
	1 vs. 2	1 vs. 3	1 vs. 4	2 vs. 3	2 vs. 4	3 vs. 4
Area	0.5636	0.9833	0.9952	0.9805	0.9935	0.6244
Std. Error	0.07062	0.01502	0.006416	0.01477	0.006852	0.1056
95% confidence interval	0.4252 to 0.7020	0.9539 to 1.000	0.9827 to 1.000	0.9515 to 1.000	0.9801 to 1.000	0.4174 to 0.8315
p-Value	0.3724	<0.0001*	<0.0001*	<0.0001*	<0.0001*	0.2455
	TLR-4+ on intermediate monocytes[%]					
	1 vs. 2	1 vs. 3	1 vs. 4	2 vs. 3	2 vs. 4	3 vs. 4
Area	0.5096	0.9952	1	0.9967	1	0.6244
Std. Error	0.07256	0.006416	0	0.004401	0	0.1056
95% confidence interval	0.3674 to 0.6518	0.9827 to 1.000	1.000 to 1.000	0.9881 to 1.000	1.000 to 1.000	0.4174 to 0.8315
p-Value	0.8931	<0.0001*	<0.0001*	<0.0001*	<0.0001*	0.2455

	TLR-3+ on non-classical monocytes[%]					
	1 vs. 2	1 vs. 3	1 vs. 4	2 vs. 3	2 vs. 4	3 vs. 4
Area	0.5044	0.9143	0.9429	0.9366	0.974	0.6244
Std. Error	0.07199	0.04344	0.03697	0.03106	0.01741	0.1056
95% confidence interval	0.3633 to 0.6454	0.8291 to 0.9994	0.8704 to 1.000	0.8757 to 0.9975	0.9399 to 1.000	0.4174 to 0.8315
p-Value	0.9513	<0.0001*	<0.0001*	<0.0001*	<0.0001*	0.2455
	TLR-9+ on non-classical monocytes[%]					
	1 vs. 2	1 vs. 3	1 vs. 4	2 vs. 3	2 vs. 4	3 vs. 4
Area	0.5004	0.9738	0.9905	0.9821	0.9951	0.6244
Std. Error	0.07238	0.02053	0.01096	0.01349	0.005571	0.1056
95% confidence interval	0.3586 to 0.6423	0.9336 to 1.000	0.9690 to 1.000	0.9557 to 1.000	0.9842 to 1.000	0.4174 to 0.8315
p-Value	0.9951	<0.0001*	<0.0001*	<0.0001*	<0.0001*	0.2455
	TLR-2+ on non-classical monocytes[%]					
	1 vs. 2	1 vs. 3	1 vs. 4	2 vs. 3	2 vs. 4	3 vs. 4
Area	0.5017	1	1	1	1	0.6244
Std. Error	0.07238	0	0	0	0	0.1056
95% confidence interval	0.3599 to 0.6436	1.000 to 1.000	1.000 to 1.000	1.000 to 1.000	1.000 to 1.000	0.4174 to 0.8315
p-Value	0.9805	<0.0001*	<0.0001*	<0.0001*	<0.0001*	0.2455
	TLR-4+ on non-classical monocytes[%]					
	1 vs. 2	1 vs. 3	1 vs. 4	2 vs. 3	2 vs. 4	3 vs. 4
Area	0.5283	1	1	1	1	0.6244
Std. Error	0.07146	0	0	0	0	0.1056
95% confidence interval	0.3883 to 0.6684	1.000 to 1.000	1.000 to 1.000	1.000 to 1.000	1.000 to 1.000	0.4174 to 0.8315
p-Value	0.6913	<0.0001*	<0.0001*	<0.0001*	<0.0001*	0.2455
	Serum concentration of sTLR-2					
	1 vs. 2	1 vs. 3	1 vs. 4	2 vs. 3	2 vs. 4	3 vs. 4

Area	0.5296	1	1	1	1	0.6178
Std. Error	0.07133	0	0	0	0	0.1061
95% confidence interval	0.3898 to 0.6694	1.000 to 1.000	1.000 to 1.000	1.000 to 1.000	1.000 to 1.000	0.4099 to 0.8256
p-Value	0.6778	<0.0001*	<0.0001*	<0.0001*	<0.0001*	0.2717
	Serum concentration of sTLR-4					
	1 vs. 2	1 vs. 3	1 vs. 4	2 vs. 3	2 vs. 4	3 vs. 4
Area	0.5531	1	1	1	1	0.5289
Std. Error	0.0721	0	0	0	0	0.111
95% confidence interval	0.4118 to 0.6945	1.000 to 1.000	1.000 to 1.000	1.000 to 1.000	1.000 to 1.000	0.3114 to 0.7464
p-Value	0.456	<0.0001*	<0.0001*	<0.0001*	<0.0001*	0.7875
	Serum concentration of sTLR-3					
	1 vs. 2	1 vs. 3	1 vs. 4	2 vs. 3	2 vs. 4	3 vs. 4
Area	0.5192	1	1	1	1	0.5822
Std. Error	0.07106	0	0	0	0	0.1069
95% confidence interval	0.3799 to 0.6584	1.000 to 1.000	1.000 to 1.000	1.000 to 1.000	1.000 to 1.000	0.3727 to 0.7917
p-Value	0.7881	<0.0001*	<0.0001*	<0.0001*	<0.0001*	0.4429
	Serum concentration of sTLR-9					
	1 vs. 2	1 vs. 3	1 vs. 4	2 vs. 3	2 vs. 4	3 vs. 4
Area	0.5453	1	1	1	1	0.5267
Std. Error	0.07249	0	0	0	0	0.1095
95% confidence interval	0.4032 to 0.6874	1.000 to 1.000	1.000 to 1.000	1.000 to 1.000	1.000 to 1.000	0.3121 to 0.7412
p-Value	0.5251	<0.0001*	<0.0001*	<0.0001*	<0.0001*	0.8035

Supplementary Materials Table S8- ROC curve analysis for age of GC and HV patients

	BDCA-1+TLR-3+[%]					
	1 vs. 2	1 vs. 3	1 vs. 4	2 vs. 3	2 vs. 4	3 vs. 4
Area	0.7772	0.8634	0.9152	0.6349	0.6556	0.5079
Std. Error	0.05732	0.05222	0.04538	0.08161	0.09212	0.1123
95% confidence interval	0.6648 to 0.8895	0.7611 to 0.9658	0.8263 to 1.000	0.4750 to 0.7949	0.4750 to 0.8361	0.2879 to 0.7280
p-Value	<0.0001*	<0.0001*	0.0001*	0.1038	0.1615	0.9459
	BDCA-1+TLR-9+[%]					
	1 vs. 2	1 vs. 3	1 vs. 4	2 vs. 3	2 vs. 4	3 vs. 4
Area	0.7772	0.9649	1	0.8048	0.8833	0.5291
Std. Error	0.05732	0.02143	0	0.06088	0.0622	0.107
95% confidence interval	0.6648 to 0.8895	0.9229 to 1.000	1.000 to 1.000	0.6854 to 0.9241	0.7614 to 1.000	0.3193 to 0.7389
p-Value	<0.0001*	<0.0001*	<0.0001*	0.0002*	0.0006*	0.8034
	BDCA-1+TLR-2+[%]					
	1 vs. 2	1 vs. 3	1 vs. 4	2 vs. 3	2 vs. 4	3 vs. 4
Area	0.7781	1	1	1	1	0.5053
Std. Error	0.05723	0	0	0	0	0.1278
95% confidence interval	0.6659 to 0.8902	1.000 to 1.000	1.000 to 1.000	1.000 to 1.000	1.000 to 1.000	0.2548 to 0.7558
p-Value	<0.0001*	<0.0001*	<0.0001*	<0.0001*	<0.0001*	0.9639
	BDCA-1+TLR-4+[%]					
	1 vs. 2	1 vs. 3	1 vs. 4	2 vs. 3	2 vs. 4	3 vs. 4
Area	0.7768	1	1	0.9921	0.9963	0.5132
Std. Error	0.05737	0	0	0.007801	0.006102	0.1166
95% confidence interval	0.6643 to 0.8892	1.000 to 1.000	1.000 to 1.000	0.9768 to 1.000	0.9843 to 1.000	0.2847 to 0.7417
p-Value	<0.0001*	<0.0001*	<0.0001*	<0.0001*	<0.0001*	0.9099
	BDCA-2+TLR-3+[%]					

	1 vs. 2	1 vs. 3	1 vs. 4	2 vs. 3	2 vs. 4	3 vs. 4
Area	0.7772	0.9355	0.8918	0.6508	0.6037	0.6032
Std. Error	0.05732	0.03543	0.05667	0.07762	0.09499	0.1173
95% confidence interval	0.6648 to 0.8895	0.8660 to 1.000	0.7807 to 1.000	0.4987 to 0.8029	0.4175 to 0.7899	0.3733 to 0.8330
p-Value	<0.0001*	<0.0001*	0.0003*	0.069	0.3506	0.3775
	BDCA-2+TLR-9+[%]					
	1 vs. 2	1 vs. 3	1 vs. 4	2 vs. 3	2 vs. 4	3 vs. 4
Area	0.7768	1	1	0.9238	0.9074	0.6032
Std. Error	0.05735	0	0	0.03584	0.04691	0.1173
95% confidence interval	0.6644 to 0.8892	1.000 to 1.000	1.000 to 1.000	0.8536 to 0.9940	0.8155 to 0.9993	0.3733 to 0.8330
p-Value	<0.0001*	<0.0001*	<0.0001*	<0.0001*	0.0002*	0.3775
	BDCA-2+TLR-2+[%]					
	1 vs. 2	1 vs. 3	1 vs. 4	2 vs. 3	2 vs. 4	3 vs. 4
Area	0.7772	1	1	0.9889	0.9852	0.6032
Std. Error	0.05729	0	0	0.009996	0.01516	0.1173
95% confidence interval	0.6649 to 0.8895	1.000 to 1.000	1.000 to 1.000	0.9693 to 1.000	0.9555 to 1.000	0.3733 to 0.8330
p-Value	<0.0001*	<0.0001*	<0.0001*	<0.0001*	<0.0001*	0.3775
	BDCA-2+TLR-4+[%]					
	1 vs. 2	1 vs. 3	1 vs. 4	2 vs. 3	2 vs. 4	3 vs. 4
Area	0.7768	1	1	1	1	0.6032
Std. Error	0.05734	0	0	0	0	0.1173
95% confidence interval	0.6644 to 0.8891	1.000 to 1.000	1.000 to 1.000	1.000 to 1.000	1.000 to 1.000	0.3733 to 0.8330
p-Value	<0.0001*	<0.0001*	<0.0001*	<0.0001*	<0.0001*	0.3775
	TLR-3+ on classical monocytes[%]					
	1 vs. 2	1 vs. 3	1 vs. 4	2 vs. 3	2 vs. 4	3 vs. 4
Area	0.7689	0.6353	0.5599	0.6643	0.687	0.6032

Std. Error	0.05798	0.07305	0.1064	0.07949	0.1216	0.1321
95% confidence interval	0.6552 to 0.8825	0.4922 to 0.7785	0.3515 to 0.7684	0.5085 to 0.8201	0.4488 to 0.9253	0.3442 to 0.8621
p-Value	0.0002*	0.0873	0.5794	0.0476*	0.0923	0.3775
	TLR-9+ on classical monocytes [%]					
	1 vs. 2	1 vs. 3	1 vs. 4	2 vs. 3	2 vs. 4	3 vs. 4
Area	0.7772	0.8064	0.8187	0.5119	0.5148	0.545
Std. Error	0.05732	0.05527	0.063	0.08279	0.09073	0.1149
95% confidence interval	0.6648 to 0.8895	0.6981 to 0.9147	0.6952 to 0.9422	0.3496 to 0.6742	0.3370 to 0.6926	0.3197 to 0.7702
p-Value	<0.0001*	0.0001*	0.0032*	0.8859	0.8939	0.7005
	TLR-2+ on classical monocytes[%]					
	1 vs. 2	1 vs. 3	1 vs. 4	2 vs. 3	2 vs. 4	3 vs. 4
Area	0.7684	1	1	0.9762	0.9889	0.545
Std. Error	0.05829	0	0	0.01713	0.01341	0.1149
95% confidence interval	0.6542 to 0.8827	1.000 to 1.000	1.000 to 1.000	0.9426 to 1.000	0.9626 to 1.000	0.3197 to 0.7702
p-Value	0.0002*	<0.0001*	<0.0001*	<0.0001*	<0.0001*	0.7005
	TLR-4+ on classical monocytes [%]					
	1 vs. 2	1 vs. 3	1 vs. 4	2 vs. 3	2 vs. 4	3 vs. 4
Area	0.7768	1	1	0.9175	0.9481	0.545
Std. Error	0.05734	0	0	0.03788	0.03473	0.1149
95% confidence interval	0.6644 to 0.8891	1.000 to 1.000	1.000 to 1.000	0.8432 to 0.9917	0.8801 to 1.000	0.3197 to 0.7702
p-Value	<0.0001*	<0.0001*	<0.0001*	<0.0001*	<0.0001*	0.7005
	TLR-3+ on intermediate monocytes[%]					
	1 vs. 2	1 vs. 3	1 vs. 4	2 vs. 3	2 vs. 4	3 vs. 4
Area	0.5474	0.8596	0.8947	0.8857	0.9315	0.5423
Std. Error	0.07094	0.04796	0.04781	0.05041	0.04252	0.1128

95% confidence interval	0.4083 to 0.6864	0.7657 to 0.9536	0.8010 to 0.9884	0.7869 to 0.9845	0.8481 to 1.000	0.3212 to 0.7635
p-Value	0.5048	<0.0001*	0.0003*	<0.0001*	0.0001*	0.7173
	TLR-9+ on intermediate monocytes [%]					
	1 vs. 2	1 vs. 3	1 vs. 4	2 vs. 3	2 vs. 4	3 vs. 4
Area	0.5219	0.8772	0.9152	0.8698	0.9074	0.5423
Std. Error	0.07183	0.04425	0.04115	0.05114	0.0479	0.1128
95% confidence interval	0.3811 to 0.6627	0.7905 to 0.9639	0.8346 to 0.9959	0.7696 to 0.9701	0.8135 to 1.000	0.3212 to 0.7635
p-Value	0.7575	<0.0001*	<0.0001*	<0.0001*	0.0002*	0.7173
	TLR-2+ on intermediate monocytes [%]					
	1 vs. 2	1 vs. 3	1 vs. 4	2 vs. 3	2 vs. 4	3 vs. 4
Area	0.5794	0.9762	0.9883	0.9968	1	0.5423
Std. Error	0.06964	0.01536	0.01197	0.004325	0	0.1128
95% confidence interval	0.4429 to 0.7159	0.9461 to 1.000	0.9649 to 1.000	0.9883 to 1.000	1.000 to 1.000	0.3212 to 0.7635
p-Value	0.2637	<0.0001*	<0.0001*	<0.0001*	<0.0001*	0.7173
	TLR-4+ on intermediate monocytes [%]					
	1 vs. 2	1 vs. 3	1 vs. 4	2 vs. 3	2 vs. 4	3 vs. 4
Area	0.5535	0.9975	1	0.9968	1	0.5423
Std. Error	0.07147	0.003424	0	0.004325	0	0.1128
95% confidence interval	0.4134 to 0.6936	0.9908 to 1.000	1.000 to 1.000	0.9883 to 1.000	1.000 to 1.000	0.3212 to 0.7635
p-Value	0.4512	<0.0001	<0.0001	<0.0001	<0.0001	0.7173
	TLR-3+ on non-classical monocytes [%]					
	1 vs. 2	1 vs. 3	1 vs. 4	2 vs. 3	2 vs. 4	3 vs. 4
Area	0.7531	0.9925	1	0.8683	0.9037	0.5423
Std. Error	0.06	0.007552	0	0.04974	0.0489	0.1128
95% confidence interval	0.6355 to 0.8707	0.9777 to 1.000	1.000 to 1.000	0.7708 to 0.9657	0.8079 to 0.9995	0.3212 to 0.7635

p-Value	0.0004*	<0.0001*	<0.0001*	<0.0001*	0.0003*	0.7173
	TLR-9+ on non-classical monocytes [%]					
	1 vs. 2	1 vs. 3	1 vs. 4	2 vs. 3	2 vs. 4	3 vs. 4
Area	0.7482	1	1	0.9619	0.9815	0.5423
Std. Error	0.06101	0	0	0.02303	0.01739	0.1128
95% confidence interval	0.6287 to 0.8678	1.000 to 1.000	1.000 to 1.000	0.9168 to 1.000	0.9474 to 1.000	0.3212 to 0.7635
p-Value	0.0005*	<0.0001*	<0.0001*	<0.0001*	<0.0001*	0.7173
	TLR-2+ on non-classical monocytes[%]					
	1 vs. 2	1 vs. 3	1 vs. 4	2 vs. 3	2 vs. 4	3 vs. 4
Area	0.7487	1	1	1	1	0.5423
Std. Error	0.06089	0	0	0	0	0.1128
95% confidence interval	0.6293 to 0.8680	1.000 to 1.000	1.000 to 1.000	1.000 to 1.000	1.000 to 1.000	0.3212 to 0.7635
p-Value	0.0005*	<0.0001*	<0.0001*	<0.0001*	<0.0001*	0.7173
	TLR-4+ on non-classical monocytes[%]					
	1 vs. 2	1 vs. 3	1 vs. 4	2 vs. 3	2 vs. 4	3 vs. 4
Area	0.7772	1	1	1	1	0.5423
Std. Error	0.05642	0	0	0	0	0.1128
95% confidence interval	0.6666 to 0.8878	1.000 to 1.000	1.000 to 1.000	1.000 to 1.000	1.000 to 1.000	0.3212 to 0.7635
p-Value	<0.0001*	<0.0001*	<0.0001*	<0.0001*	<0.0001*	0.7173
	Serum concentration of sTLR-2					
	1 vs. 2	1 vs. 3	1 vs. 4	2 vs. 3	2 vs. 4	3 vs. 4
Area	0.557	1	1	1	1	0.5982
Std. Error	0.07082	0	0	0	0	0.1275
95% confidence interval	0.4182 to 0.6958	1.000 to 1.000	1.000 to 1.000	1.000 to 1.000	1.000 to 1.000	0.3484 to 0.8480
p-Value	0.4238	<0.0001*	<0.0001*	<0.0001*	<0.0001*	0.4208

	Serum concentration of sTLR-4					
	1 vs. 2	1 vs. 3	1 vs. 4	2 vs. 3	2 vs. 4	3 vs. 4
Area	0.5871	1	1	1	1	0.6667
Std. Error	0.06944	0	0	0	0	0.09825
95% confidence interval	0.4510 to 0.7232	1.000 to 1.000	1.000 to 1.000	1.000 to 1.000	1.000 to 1.000	0.4741 to 0.8592
p-Value	0.2219	<0.0001*	<0.0001*	<0.0001*	<0.0001*	0.1719
	Serum concentration of sTLR-3					
	1 vs. 2	1 vs. 3	1 vs. 4	2 vs. 3	2 vs. 4	3 vs. 4
Area	0.5703	1	1	1	1	0.622
Std. Error	0.07021	0	0	0	0	0.1198
95% confidence interval	0.4327 to 0.7079	1.000 to 1.000	1.000 to 1.000	1.000 to 1.000	1.000 to 1.000	0.3873 to 0.8568
p-Value	0.3242	<0.0001*	<0.0001*	<0.0001*	<0.0001*	0.3172
	Serum concentration of sTLR-9					
	1 vs. 2	1 vs. 3	1 vs. 4	2 vs. 3	2 vs. 4	3 vs. 4
Area	0.588	1	1	1	1	0.622
Std. Error	0.06949	0	0	0	0	0.1331
95% confidence interval	0.4518 to 0.7242	1.000 to 1.000	1.000 to 1.000	1.000 to 1.000	1.000 to 1.000	0.3612 to 0.8829
p-Value	0.2173	<0.0001*	<0.0001*	<0.0001*	<0.0001*	0.3172

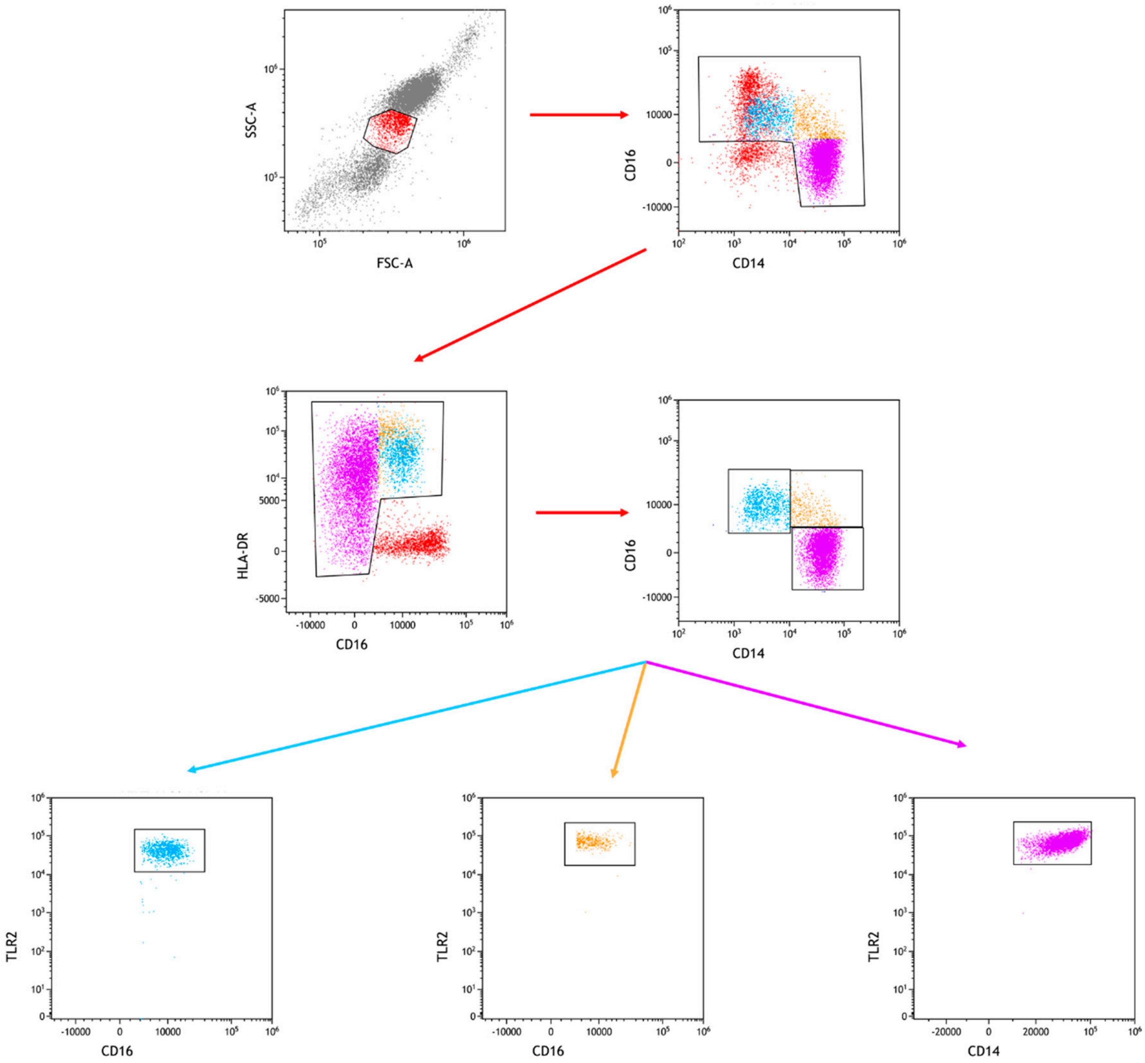

Supplementary Materials Figure S1 - Example Analysis of Monocyte Subpopulations in Peripheral Blood

Flow flow cytometric analysis of peripheral blood monocyte subpopulations was performed using CD14, CD16, and HLA-DR markers. Initially, monocytes were identified based on their physical properties using the FSC-A vs SSC-A plot, which allowed exclusion of contaminants and uninteresting cell populations. Monocytes were then divided into subpopulations based on CD14, CD16, and HLA-DR expression.
